# Effect of porosity on catalytic performance of HKUST-1 in Knoevenagel condensation

**DOI:** 10.1038/s41598-026-59395-w

**Published:** 2026-06-23

**Authors:** Nikola Vargová, Milica Želinská, Ľuboš Zauška, Lucie Zelená, Rastislav Serbin, Daria Striežovská, Jozef Bednarčík, Tomáš Zelenka, Marta Férová, Peter Obšatník, Vladimír Zeleňák, Miroslav Almáši, Nikolas Király

**Affiliations:** 1 Department of Inorganic Chemistry, Institute of Chemistry, Faculty of Science, Pavol Jozef Šafárik University in Košice, Moyzesova 11, Košice, 041 54 Slovakia; 2 Department of Chemistry, Faculty of Science, University of Ostrava, 30. Dubna 22, Ostrava, 702 00 Czech Republic; 3 Department of Analytical Chemistry, Institute of Chemistry, Faculty of Science, Pavol Jozef Šafárik University in Košice, Moyzesova 11, Košice, 041 54 Slovakia; 4 Department of Condensed Matter Physics, Institute of Physics, Faculty of Science, Pavol Jozef Šafárik University in Košice, Moyzesova 11, Košice, 041 54 Slovakia

**Keywords:** Metal–organic frameworks, MOF, HKUST-1, Knoevenagel condensation, Heterogeneous catalysis, Chemistry, Materials science

## Abstract

Hierarchically porous HKUST-1 materials (**A** and **B**) were synthesized via a cooperative template-directed strategy enabling controlled introduction of mesoporosity while preserving the characteristic original framework (**1**). Structural, spectroscopic, and textural characterization confirmed retention of the intrinsic HKUST-1 structure together with high specific surface areas of 1788, 1554, and 1690 m^2^ g^− 1^ for activated **1’**, **A’**, and **B’**, respectively. The hierarchical materials exhibited pore size distributions extending from intrinsic micropores to mesopores. The catalytic performance was evaluated in the Knoevenagel condensation of benzaldehyde derivatives with malononitrile. Under optimized conditions (toluene, 80°C, 50 mg catalyst, 60 min), conversions of 93% and 98% were achieved for **A’** and **B’**, respectively. Arrhenius analysis yielded a lower effective activation energy for **B’** (43.9 ± 5.2 kJ mol^− 1^) compared to **A’** (53.6 ± 3.1 kJ mol^− 1^), indicating more favorable reaction kinetics. High conversions (~ 96–98%) were obtained for electron-withdrawing substituents, whereas bulky alkyl-substituted substrates showed lower conversions (~ 6–16% for **B’**, ~ 5–11% for **A’**), confirming diffusion limitations. Recyclability tests showed only moderate activity loss after five cycles (17% for **A’**, 15% for **B’**). Post-catalysis analysis confirmed preserved framework structure and crystallinity, while infrared and thermogravimetric analyses indicate residual species in the pores.

**Supplementary Information:**

The online version contains supplementary material available at https://doi.org/10.1038/s41598-026-59395-w.

## Introduction

Metal–organic frameworks (MOFs) constitute a class of crystalline porous materials formed through coordination bonding between metal ions or clusters and multitopic organic ligands, offering exceptionally high surface areas, structural tunability, and chemically addressable active sites^1,2^. Owing to their modular construction, MOFs enable precise control over pore size, topology, and chemical functionality, which has positioned them as highly promising materials for applications in gas storage, separation, sensing, water harvesting, and heterogeneous catalysis^3–6^. Despite these advantages, most conventional MOFs are intrinsically microporous, with pore diameters typically below 2 nm, which can impose severe diffusion limitations for bulky reactants and products under catalytic conditions^7^. In such systems, restricted mass transport frequently leads to reduced apparent reaction rates, incomplete utilization of internal active sites, and an increased susceptibility to pore blocking during catalytic turnover^8–10^. Consequently, the rational introduction of mesoporosity into MOF architectures has emerged as an effective strategy to mitigate diffusion constraints by shortening transport pathways, improving reactant accessibility, and enabling more efficient utilization of catalytically active centers^11,12^.

Among the vast family of MOFs, HKUST-1, also known as CuBTC or MOF-199, remains one of the most extensively studied benchmark materials due to its well-defined structure, chemical robustness, and catalytic versatility^13,14^. HKUST-1 is constructed from [Cu(COO)_4_(H_2_O)_2_] paddle-wheel secondary building units (SBUs) linked by 1,3,5-benzenetricarboxylate linkers to form a three-dimensional framework exhibiting high specific surface areas typically in the range of 1500–1800 m^2^ g^-1^. The structure contains two distinct microporous domains consisting of large cages with diameters of ~ 0.9–1.1 nm and smaller cages interconnected through microporous windows with apertures of ~ 0.35–0.45 nm^15^. Upon removal of coordinated water molecules by thermal or vacuum activation, each copper paddle-wheel SBU exposes two coordinatively unsaturated sites (CUSs), which act as Lewis acidic centers capable of strongly interacting with polar functional groups^16^. Importantly, the copper centers can undergo reversible Cu(II)/Cu(I) redox transitions, which further extends the applicability of HKUST-1 to redox-driven heterogeneous catalytic processes in addition to Lewis acid catalysis^17–19^. CUSs are widely recognized as the primary origin of HKUST-1 catalytic activity in a broad range of organic transformations, including oxidation reactions, acylations, condensations, and multicomponent coupling reactions^20–24^. In carbon–carbon bond-forming reactions, such as the Knoevenagel condensation, HKUST-1 facilitates adsorption and activation of carbonyl substrates while stabilizing reaction intermediates within its confined pore environment^25^. The Knoevenagel condensation represents one of the most widely studied carbon–carbon bond-forming transformations between aldehydes or ketones and active methylene compounds, such as malononitrile or ethyl cyanoacetate, and is widely applied in the synthesis of fine chemicals, pharmaceuticals, agrochemicals, dyes, and functional materials^26,27^. However, despite the promising catalytic properties of HKUST-1, purely microporous frameworks often exhibit reduced catalytic efficiency toward sterically demanding substrates due to slow intracrystalline diffusion and limited accessibility of internal active sites^28^. From a broader catalytic perspective, traditional homogeneous systems used for Knoevenagel-type transformations, typically based on amines or metal salts, often suffer from drawbacks such as difficult separation, limited recyclability, and environmental concerns^29–31^. In this context, heterogeneous catalysts based on porous solids have emerged as attractive alternatives, as they enable efficient catalyst recovery and reuse while improving overall process sustainability^32–34^.

To overcome the intrinsic mass transport limitations associated with purely microporous MOFs, significant research efforts have focused on the development of hierarchical MOF architectures integrating both micropores and mesopores within a single crystalline framework^35–38^. Such hierarchical structures aim to preserve the high density of catalytically active sites characteristic of microporous MOFs while simultaneously improving molecular diffusion through the introduction of mesoporous transport pathways. Unlike conventional mesoporous oxides or silicas, however, the incorporation of mesoporosity into MOFs remains particularly challenging due to the delicate balance required between metal–ligand coordination chemistry and templating interactions during crystallization^39^. Early attempts based solely on surfactant templating were largely unsuccessful, as surfactants frequently interfere with framework nucleation and growth, leading to amorphous products or phase separation^40^. A breakthrough was achieved with cooperative template-directed assembly strategies, which utilize both surfactant micelles and chelating or bridging agents to simultaneously control mesostructure formation and coordination-driven framework crystallization^41,42^. Within this cooperative strategy, the surfactant defines mesoscopic domains, while the chelating agent regulates metal ion availability and coordination kinetics, enabling the formation of ordered MOF structures around the template. The application of this concept to HKUST-1 has enabled the preparation of hierarchical micro–mesoporous frameworks that retain the intrinsic crystallinity and coordination environment of the parent material. Importantly, the mesopore size and volume can be systematically tuned by adjusting the surfactant concentration and the ratio between templating and chelating components. As a result, hierarchical HKUST-1 materials combine the high density of catalytically active sites typical of microporous MOFs with significantly improved mass transport properties arising from mesoporous channels^43,44^.

In this work, hierarchically porous HKUST-1 materials are synthesized via a cooperative template-directed strategy to introduce mesoporosity while preserving the characteristic framework. The resulting materials are comprehensively characterized with respect to structural integrity, porosity, and stability, and their catalytic performance is evaluated in the Knoevenagel condensation of benzaldehyde derivatives and malononitrile.

## Experimental

### Materials and methods

All chemicals used for the synthesis of the materials and for catalytic testing were purchased from commercial suppliers (eMolecules, Acros Organics, and Sigma-Aldrich) and used as received without further purification.

Infrared spectra were recorded using a Thermo Scientific Nicolet 6700 FT-IR spectrometer (Thermo Fisher Scientific, USA) in the wavenumber range of 4000–400 cm^− 1^ with a spectral resolution of 4 cm^− 1^ and 128 scans per spectrum. Measurements were performed using the ATR technique with a SmartOrbit™ accessory.

Powder X-ray diffraction (PXRD) measurements were performed in reflection geometry using a Bruker D2 Phaser diffractometer (Bruker, Germany) equipped with a Cu Kα radiation source (*λ* = 1.5406 Å). The instrument was operated using a Ni-filtered Cu Kα radiation and standard Bragg–Brentano geometry. PXRD experiments were conducted by 2*θ* continuous scans from 5 to 30° with a measurement speed of 0.5 min^− 1^, and a NaI scintillation detector was used for the recording of diffracted photons.

The textural properties of the prepared samples were assessed based on the adsorption/desorption isotherms of nitrogen at -196 °C, similarly to^45,46^. Before the adsorption measurements, solvent molecules were removed from the porous structure by degassing at room temperature for 2 h, then at 60 °C for another 2 h, and finally subjected to vacuum degassing on the adsorption analyzer for 16 h at 100 °C. Adsorption isotherms were measured using a Quantachrome Autosorb-iQ-XR. The analysis and evaluation of the adsorption isotherms and properties were performed using the ASiQwin software from Quantachrome Instruments. The N_2_ adsorption isotherms were used to calculate the BET area (*S*_*BET*_) with respect to Rouquerol’s consistency criteria^47,48^. The volume and surface area of micropores (pore diameter < 2 nm) and mesopores (pore diameter 2–50 nm), as well as total pore volume and surface area (pore diameter < 50 nm), were calculated from pore size distribution (PSD) curves. These were obtained by fitting the experimental adsorption data with a Non-Local Density Functional Theory (NLDFT) adsorption kernel (a set of theoretical isotherms; ASiQwin software, Quantachrome Instruments), assuming a cylindrical pore shape.

Reaction progress, including substrate conversion and product selectivity, was determined by gas chromatography (GC) using a Shimadzu Nexis GC-2030 instrument (Shimadzu Corporation, Japan) equipped with an AOC-20i Plus autosampler. Chromatographic separation was performed on an SH-Rxi-5ms capillary column (30 m × 0.25 mm i.d., 0.25 μm film thickness) coupled with an Rtx-1701 guard column with Integra-Guard (30 m × 0.25 mm, 0.25 μm film thickness). Nitrogen was used as the carrier gas with a linear velocity of 50 cm s^-1^ and was also employed as the detector make-up gas, corresponding to a column flow rate of approximately 2.44 mL min^-1^. The injector temperature was set to 250 °C with a split ratio of 50:1, while the detector temperature was maintained at 280 °C. The flow rates of hydrogen, air, and nitrogen make-up gas were set to 32, 200, and 24 mL min^-1^, respectively. The oven temperature program was initiated at 70 °C, followed by a ramp to 270 °C at a rate of 25 °C min^-1^ and a final hold time of 7 min, giving a total run time of 15 min. The injection volume was 1 µL for all analyses.

Particle size determination was performed using dynamic light scattering (DLS) under conditions corresponding to the catalytic experiments. The measurements were carried out on a Zetasizer Advance Ultra (red, Malvern Instruments, UK) and ZS Xplorer software version 4.2.0 (Malvern Instruments, UK) using a glass cuvette with a square aperture (PCS1115). For the analysis, 50 mg of activated catalysts, pretreated at 120 °C for 60 min, were dispersed in 50 mL of toluene. Prior to measurement, the suspensions were ultrasonicated in a water bath for 5 min (SONOREX Digital 10P type DK102P, BANDELIN electronic, Germany) to break up any agglomerates and to ensure proper particle dispersion. The instrument operates using a He–Ne laser (*λ* = 632.8 nm) with backscatter detection at an angle of 173°, enabling determination of the hydrodynamic particle size distribution of the dispersed particles. Each sample was measured several times, and the results provided are the average of three measurements.

Thermal properties of the prepared materials and samples after catalysis were investigated by thermogravimetric analysis (TGA) using a STA 509 Select (NETZSCH, Germany). All samples were measured in an air atmosphere (50 mL min^-1^ over a temperature range from 30 to 900 °C with a heating rate of 10 °C min^-1^.

Atomic absorption spectroscopy (AAS) measurements were performed using a PerkinElmer Analyst 200 spectrometer (PerkinElmer, USA) to determine the possible leaching of Cu(II) cations from the catalyst into the reaction solution. The analyses were carried out in flame mode using an air–acetylene flame at the characteristic Cu resonance line. Under the applied experimental conditions, the detection limit for Cu(II) was 0.005 mg L^− 1^.

Column chromatography was performed using silica gel (Silicagel A60, 0.040–0.063 mm, 230–400 mesh, Centralchem). Thin-layer chromatography (TLC) was carried out using ALUGRAM^®^ SIL G/UV254 plates (Macherey-Nagel GmbH & Co), and spots were visualized under UV light (254 nm).

NMR spectra were recorded on a Varian Mercury Plus 400 FT NMR (400.13 MHz for ^1^H and 100.61 MHz for ^13^C) spectrometer (Agilent Technologies, USA). Chemical shift values *δ* are given in parts per million (ppm) relative to the solvent signals for ^1^H relative to CDCl_3_ (7.26 ppm), for ^13^C relative to CDCl_3_ (77.00 ppm) and coupling constants were recorded in Hertz. The multiplicity of the ^13^C NMR signals arising from ^13^C to ^1^H coupling was determined by HSQC experiments. Chemical shifts (ppm) and coupling constants (Hz) were obtained by first-order spectral analysis. Signal assignments were achieved on the basis of homonuclear ^1^H–^1^H COSY and heteronuclear ^1^H–^13^C 2D correlation spectra (HSQC, HMBC).

### Synthesis of catalysts

HKUST-1 (**1**) was synthesized via a conventional solvothermal method by dissolving trimesic acid (H₃BTC, 1.00 g, 4.76 mmol) in 30 mL of an ethanol/*N*,* N*-dimethylformamide (EtOH/DMF, 1:1 v/v) mixture^49^. In a separate vessel, Cu(NO_3_)_2_·3H_2_O (2.08 g, 8.61 mmol) was dissolved in 15 mL of H_2_O. The two solutions were combined, and the resulting reaction mixture was heated at 85 °C for 24 h. The resulting blue crystalline solid was collected by filtration and washed with EtOH. Solvent exchange was subsequently carried out by immersing the as-synthesized material (AS) in methanol for 5 days to replace guest solvent molecules located within the pore structure. During this period, the methanol was decanted and replaced with fresh solvent every 24 h. The material after the solvent exchange process is designated in the text as (**1**).

Hierarchically structured catalysts **A** and **B** were synthesized using a cooperative templating strategy for the preparation of mesostructured MOFs. The synthesis procedure was adapted from the method reported in^44^. A mixture of trimesic acid (H_3_BTC, 116 mg, 0.56 mmol) and Cu(NO_3_)_2_·3H_2_O (242 mg, 1.00 mmol) was dissolved in 10 mL of DMF. Subsequently, cetyltrimethylammonium bromide (CTAB) and citric acid (CA) were added as structure-directing and chelating agents, respectively. For the synthesis of **A (AS)**, 200 mg of CTAB and 20 mg of citric acid were used, whereas for **B (AS)**, 400 mg of CTAB and 50 mg of citric acid were applied. The reaction mixture was homogenized by ultrasonication for 10 min and then heated at 75 °C for 24 h. The resulting blue powder was collected by filtration and washed with EtOH. Surfactant removal was carried out in multiple steps: first, the obtained material was treated with 45 mL of 1 M NH_4_NO_3_ solution in EtOH/H_2_O (1:2, v/v) and heated at 60 °C for 24 h. The solution was then decanted and replaced with 45 mL of EtOH/H_2_O (1:2, *V/V*), followed by stirring for 24 h at room temperature. In the final step, the materials were solvent-exchanged with methanol using a Soxhlet extractor for 3 days and designated in the text as (**A** and **B**).

### Catalytic measurement

**Fig. 1:**
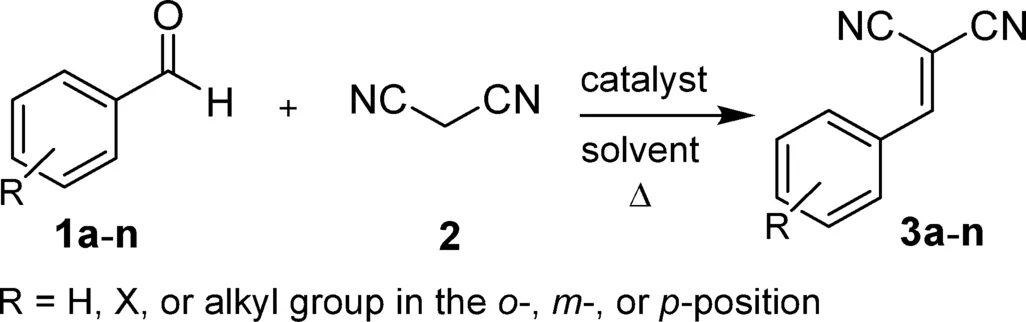
General Knoevenagel condensation of substituted aromatic aldehydes (1a–n) with malononitrile affording the corresponding benzylidenemalononitriles (3a–n).

The Knoevenagel condensation of aromatic aldehydes including benzaldehyde (1a), 2-fluorobenzaldehyde (1b), 2-chlorobenzaldehyde (1c), 2-bromobenzaldehyde (1d), 2-nitrobenzaldehyde (1e), 4-fluorobenzaldehyde (1f), 4-chlorobenzaldehyde (1 g), 4-bromobenzaldehyde (1 h), 4-nitrobenzaldehyde (1i), 2,4-difluorobenzaldehyde (1j), 2,4,6-trifluorobenzaldehyde (1k), 4-methylbenzaldehyde (1 L), 3,5-dimethylbenzaldehyde (1 m), and 4-isopropylbenzaldehyde (1n) with malononitrile was carried out in the liquid phase under atmospheric pressure at various temperatures (room temperature, 60, 80, and 100 °C), as illustrated in Fig. 1. Prior to catalytic testing, the catalysts were activated at 120 °C for 60 min to remove guest solvent molecules and generate accessible active sites. The activated samples are denoted throughout the manuscript as **1’**, **A’**, and **B’**.

In a typical experiment, 10 mL of solvent and 400 mg (4.83 mmol) of internal standard (1-bromonaphthalene) were introduced into a 25 mL two-neck round-bottom flask and heated to the desired temperature. Subsequently, 4 mmol (424 mg) of benzaldehyde or the corresponding substituted aldehyde was added. After homogenization, an aliquot (0.1 mL) was withdrawn to determine the initial composition of the reaction mixture. The activated catalyst (50 mg) was then added, followed by malononitrile (6 mmol, 396 mg), and the reaction mixture was stirred vigorously (500 rpm) at the selected temperature for the desired reaction time. Aliquots (0.1 mL) were withdrawn after 15, 30, 60, 90, 120, 150, and 180 min. After completion of the reaction, the catalyst was separated by centrifugation (4000 rpm, 10 min), and reaction products were quantified by GC using a Shimadzu Nexis GC-2030 system equipped with a FID detector. Product identities were confirmed by NMR spectroscopy (see ESI).

A blank experiment carried out using benzaldehyde and malononitrile at 80 °C in the absence of a catalyst showed no detectable product formation (0% conversion), confirming that the solvent does not promote the reaction. A leaching test was subsequently performed to evaluate catalyst stability. After centrifugation, the reaction mixture was extracted three times with deionized water. The aqueous phases were collected, concentrated and analyzed by AAS. The copper concentration in the analyzed solution was below the detection limit (0.005 mg L^− 1^). The leaching test was performed using 50 mg of catalyst, and the aqueous phase was concentrated to a final volume of 50 mL before analysis. Considering the theoretical Cu content of HKUST-1 (31.52 wt%), the maximum possible amount of leached Cu corresponds to less than 0.0016% of the total Cu present in the catalyst, confirming negligible Cu leaching and supporting the heterogeneous nature of the catalytic process.

Adsorption isotherms of benzaldehyde were measured at the two boundary temperatures used in the catalytic experiments, namely RT and 100°C. Stock solutions of benzaldehyde in toluene with concentrations of 0.05, 0.1, 0.2, 0.3, and 0.4 M were prepared. For each experiment, 50 mg of activated catalysts (**A’** and **B’**), pretreated at 120 °C for 60 min, were added to the solutions and stirred at the respective temperature (RT or 100 °C) for 180 min, corresponding to the duration of the catalytic experiments. After this period, aliquots of the solutions were collected and analyzed by GC. The amount of adsorbed benzaldehyde was calculated from the difference between the initial and final concentrations of the solution and used for evaluation of the surface coverage ($\:\theta\:$).

Catalyst recyclability was evaluated in the condensation of benzaldehyde with malononitrile over five consecutive catalytic cycles. After each cycle, the catalyst was separated by centrifugation, washed with methanol, reactivated at 120 °C for 60 min, and reused in the subsequent reaction run. The recycled catalysts were characterized by FT-IR spectroscopy and PXRD to verify structural stability.

## Results and discussion

### Structural, textural and physicochemical characterization of the catalysts

**Fig. 2:**
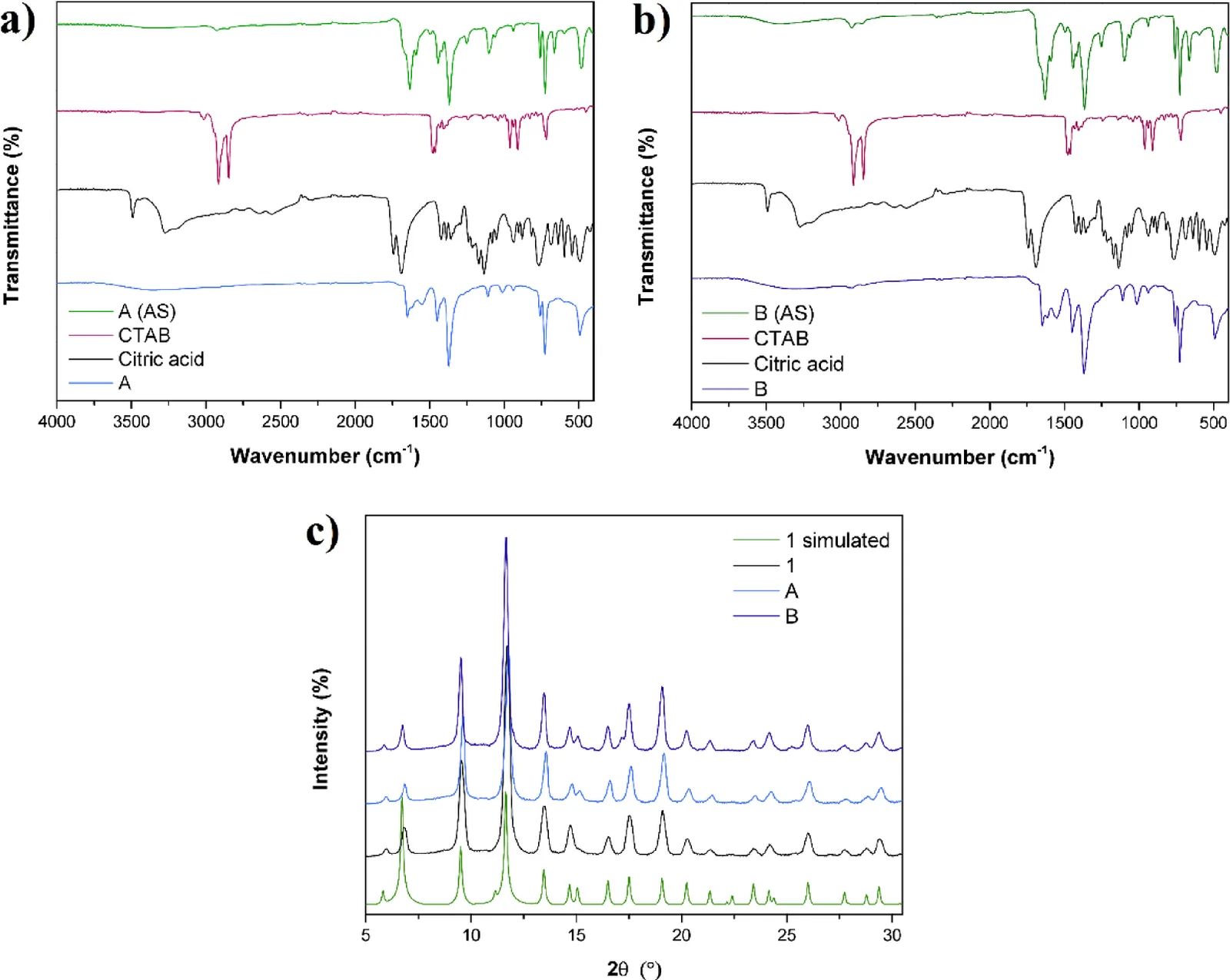
IR spectra of (**a**) **A** and (**b**) **B** before and after template removal, (**c**) PXRD patterns of simulated **1**, measured **1**, **A**, and **B**.

Infrared spectroscopy was employed to confirm the successful synthesis of the materials, verify surfactant removal, and monitor the activation process. Figure 2, Figs. S1 and S2 in ESI show the FT-IR spectra of as-synthesized HKUST-1(A) (**A (AS)**) and HKUST-1(B) (**B (AS)**) containing CTAB surfactant and citric acid, together with the spectra of the corresponding materials after removal of these species, denoted as HKUST-1(A) (**A**) and HKUST-1(B) (**B**), and activated materials HKUST-1 (**1’**), HKUST-1(A) (**A’**) and HKUST-1(B) (**B’**).

The successful formation of **A (AS)** and **B (AS)** via the cooperative templating approach is confirmed by the presence of characteristic vibrational bands associated with the trimesate linker and copper(II) paddle-wheel clusters (Fig. 2). Given the identical FT-IR features of both materials, sample **A (AS)** is discussed as a representative example. The FT-IR spectrum of **A (AS)** (Fig. 2a) exhibits a broad absorption band at 3358 cm^-1^ assigned to the *ν*(O–H) stretching vibration, indicating the presence of coordinated water molecules associated with copper centers as well as contributions from residual citric acid within the framework. The presence of CTAB surfactant is confirmed by absorption bands at 2925 and 2846 cm^-1^, corresponding to the asymmetric and symmetric stretching vibrations *ν*_*as*_(CH_2_) and *ν*_*s*_(CH_2_), respectively. Coordination of the organic ligand to copper(II) ions is evidenced by absorption bands at 1633 and 1368 cm^-1^, assigned to the asymmetric and symmetric stretching vibrations of the coordinated carboxylate groups, *ν*_as_(COO⁻) and *ν*_s_(COO⁻), respectively. This coordination is further supported by the band at 482 cm^-1^ corresponding to the *ν*(Cu–O) stretching vibration. The presence of the aromatic framework derived from the trimesate ligand is confirmed by the absorption band at 938 cm^-1^, assigned to the *δ*(C–H) deformation vibration characteristic of 1,3,5-trisubstituted aromatic rings. An additional *δ*(C–H) deformation band at 1101 cm^-1^ further supports the presence of this aromatic structural motif^50^.

The removal of the CTAB surfactant was confirmed by the disappearance of the absorption bands at 2925 and 2846 cm^-1^, corresponding to *ν*_as_(CH_2_) and *ν*_s_(CH_2_) vibrations. Similarly, the absence of the absorption band at 1250 cm-1, attributed to coupled C–O stretching and O–H bending vibrations, confirms the successful removal of citric acid from the material structure. The FT-IR spectrum of **A** (Fig. 2a) exhibits two characteristic ν(COO⁻) absorption bands at 1633 (*ν*_as_(COO⁻)) and 1368 cm^-1^(*ν*_s_(COO⁻)), confirming coordination of the trimesate ligand to Cu(II) ions. This coordination is further supported by the ν(Cu–O) stretching vibration observed at 482 cm^-1^. The presence of methanol molecules is indicated by absorption bands at 2913, 2837, and 1012 cm^-1^, corresponding to *ν*_as_(CH_3_), *ν*_s_(CH_3_), and *ν*(C–O) stretching vibrations, respectively. These bands originate from residual methanol introduced after the Soxhlet extraction procedure. The FT-IR spectrum of **B** (Fig. 2b) exhibits vibrational features consistent with those of **A**. As further demonstrated in Fig. S1 in ESI, **1**, **A**, and **B** display identical characteristic absorption bands, suggesting preservation of the key structural motifs.

Infrared spectroscopy was further employed to evaluate the activation process of the materials. Fig. S2 in ESI shows the FT-IR spectra of activated HKUST-1 materials, **1’**, **A’**, and **B’**. The successful activation of the materials is confirmed by the disappearance of the broad absorption band at approximately 3300 cm^− 1^, indicating removal of coordinated water molecules from the copper(II) centers and the formation of CUSs required for catalytic activity. Additionally, the release of encapsulated methanol molecules is evidenced by the absence of absorption bands corresponding to *ν*_as_(CH_3_), *ν*_s_(CH_3_), and *ν*(C–O) stretching vibrations at 2913, 2837, and 1012 cm^− 1^, respectively.

Figure 2c shows the PXRD patterns of **1**, **A**, and **B**, exhibiting diffraction peaks at 2*θ* values of 5.81, 6.78, 9.49, 11.74, 13.51, 14.66, 15.09, 16.52, 17.45, 19.13, 20.27, 21.40, 23.39, 24.13, 26.01, 27.66, 28.82, and 29.42°, corresponding to the (111), (200), (220), (222), (400), (331), (420), (422), (511), (440), (442), (620), (444), (711), (553), (733), (822), and (751) crystallographic planes, respectively. The close agreement of peak positions and the good match with the simulated PXRD pattern derived from the CIF of the reported HKUST-1 structure (CCDC refcode: DIHVIB)^51^, confirms that all synthesized materials are phase-pure and retain the characteristic HKUST-1 framework. Furthermore, the PXRD patterns of **A** and **B** demonstrate that high crystallinity is preserved after surfactant removal, indicating that both the cooperative templating synthesis and subsequent post-synthetic treatments do not disrupt the long-range structural order of the framework.

**Fig. 3:**
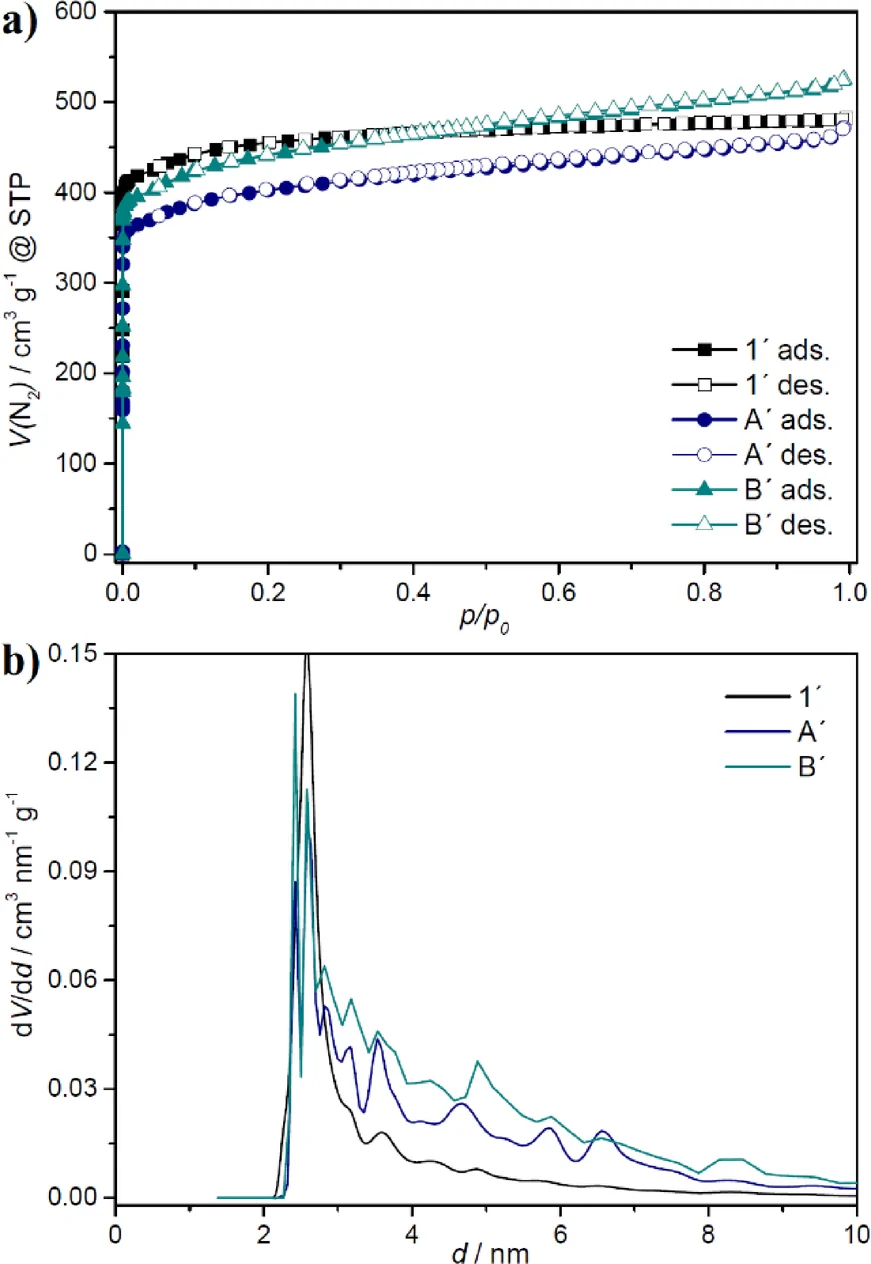
(**a**) Nitrogen adsorption/desorption isotherms and (**b**) pore size distribution curves for materials **1’**, **A’**, and **B’**.

To investigate the porosity and textural properties of the prepared materials, including BET surface area (*S*_*BET*_), pore volume (*V*_*p*_), and pore size (*d*), nitrogen adsorption/desorption measurements were performed at − 196°C (Table 1). As shown by pore size distribution (Fig. 3), the templated synthesis strategy resulted in an increased contribution of larger pores compared to parent HKUST-1, with a higher differential pore volume in the 3–9 nm range, confirming formation of hierarchical micro–mesoporous structures, while all samples also contain mesopores in the range of ca. 2.2-3 nm. Nevertheless, the presence of micropores in all samples is evidenced by their high micropore volumes and surface area values (Table 1). This limitation originates from the inadequate description of the low-pressure region by the applied NLDFT kernel (Fig. S3 in ESI). While fitting the full isotherm (10^− 6^–0.995 *p/p₀*) leads to poor agreement (fitting error 1.84%), restricting the fit to *p/p₀* ≳ 0.01 results in a substantially improved agreement (fitting error 0.15%), although at the expense of resolving the fine micropore region. The values of *S*_*BET*_ were 1788, 1554, and 1690 m^2^ g^-1^ for samples **1’**, **A’**, and **B’**, respectively. The decrease in BET area with different surfactant concentrations is consistent with partial reduction of micropore volume accompanied by formation of mesoporous domains. Analysis of volume and surface area of micropores and mesopores (Table 1) shows a decrease in micropore volume from 0.669 cm^3^ g^-1^ for **1’** to 0.554 and 0.591 cm^3^ g^-1^ for **A’** and **B’**, respectively, while mesopore volume increases from 0.084 cm^3^ g^-1^ to 0.159 and 0.210 cm^3^ g^-1^ for **A’** and **B’**, respectively. The total pore volume increased from 0.753 to 0.801 cm^3^ g^-1^ for **B’**, but slightly decreased to 0.713 cm^3^ g^-1^ for **A’**, indicating overall preservation of the porous framework. Overall, these results demonstrate that cooperative templating enables controlled introduction of mesoporosity while preserving the intrinsic HKUST-1 framework structure and high surface area, which is expected to facilitate improved mass transport during catalytic reactions.

**Table 1:** Summary of the textural properties of materials **1’**, **A’**, and **B’**.

Sample	S_BET_ (m^2^ g^− 1^)	Pore volume (cm^3^ g^− 1^)			Pore surface area (m^2^ g^− 1^)		
		Micro	Meso	Total	Micro	Meso	Total
**1’**	1788	0.669	0.084	0.753	3372	96	3468
**A’**	1554	0.554	0.159	0.713	2708	147	2854
**B’**	1690	0.591	0.210	0.801	2935	192	3127

In addition to the solid-state characterization, the dispersion behavior of the activated catalysts in the liquid phase was evaluated by dynamic light scattering (DLS) in toluene at room temperature under conditions corresponding to the catalytic experiments. The obtained results reveal relatively broad particle size distributions for all investigated samples, which is consistent with the non-uniform morphology and aggregation behavior typically observed for MOF materials dispersed in organic media. The *Z*-average values were determined to be 3677 nm for **1’**, 3196 nm for **A’**, and 2602 nm for **B’**, corresponding to the average hydrodynamic diameter (*d*_h_) of the particles in dispersion. These values are influenced by the polydispersity of the system and the presence of multiple particle size populations. The main peaks in the intensity-weighted distributions are centered at 433 nm for **1’**, 1775 nm for **A’**, and 1582 nm for **B’**. A similar trend is observed for the *d₉₀* and *d₅₀* values, indicating a consistently smaller characteristic particle size for **B’** compared to **A’** (Table 2). The relatively high polydispersity index (PDI ≈ 0.40–0.94) indicates broad particle size distributions and the presence of particle aggregates in suspension. It should be noted that the reported values correspond to hydrodynamic diameters and therefore reflect the size of dispersed aggregates rather than primary crystallite sizes. The formation of such aggregates is consistent with the known tendency of MOF particles to undergo agglomeration and partial sedimentation in organic solvents such as toluene^50,52^. Although the first peak values (*d*), *d₉₀*, and *d₅₀* for **1’** appear lower than those observed for **A’** and **B’**, this does not necessarily indicate a smaller overall particle size. The high polydispersity and pronounced aggregation tendency of **1’** likely resulted in rapid sedimentation of larger micrometer-sized aggregates during the measurement, leading to preferential detection of the remaining smaller dispersed fraction. This interpretation is consistent with the significantly higher PDI value observed for **1’** compared to the hierarchical materials.

**Table 2:** Particle size parameters of hierarchical HKUST-1 materials determined by DLS.

	d_h_ (nm)	SD*	First peak – d (nm)	d_90_ (nm)	d_50_ (nm)	Polydispersity index
**1’**	3677	534	433	503	433	0.9379
**A’**	3196	203	1775	2293	1744	0.4053
**B’**	2602	153	1582	2045	1555	0.4271

*standard deviation.

### Catalytic activity

The catalytic activity of activated materials **1’**, **A’**, and **B’** was evaluated using the Knoevenagel condensation of benzaldehyde derivatives with malononitrile to form the corresponding *α*,*β*-unsaturated nitrile products (Table 3). All Knoevenagel condensation products were synthesized and purified according to the procedures described in ESI. The structures of all isolated compounds were confirmed by ^1^H and^13^ C NMR spectroscopy, and the corresponding spectra are provided in ESI. The observed chemical shifts, coupling patterns, and signal multiplicities are fully consistent with the proposed molecular structures and, for known compounds, agree with previously reported literature data. Newly prepared derivatives were additionally characterized using 2D NMR techniques (COSY, HSQC, and HMBC) to unambiguously confirm their structural assignments. Importantly, all reactions exhibited complete (100%) selectivity toward the desired products. GC analysis revealed no detectable formation of side products, indicating excellent chemoselectivity of the catalytic system under the applied reaction conditions.

The initial stage of the catalytic study focused on optimization of reaction parameters, including solvent, temperature, catalyst loading, and reaction time. These optimization experiments were performed using malononitrile and benzaldehyde as the model carbonyl substrate, while substituted benzaldehyde derivatives were investigated subsequently under the optimized reaction conditions.

**Fig. 4:**
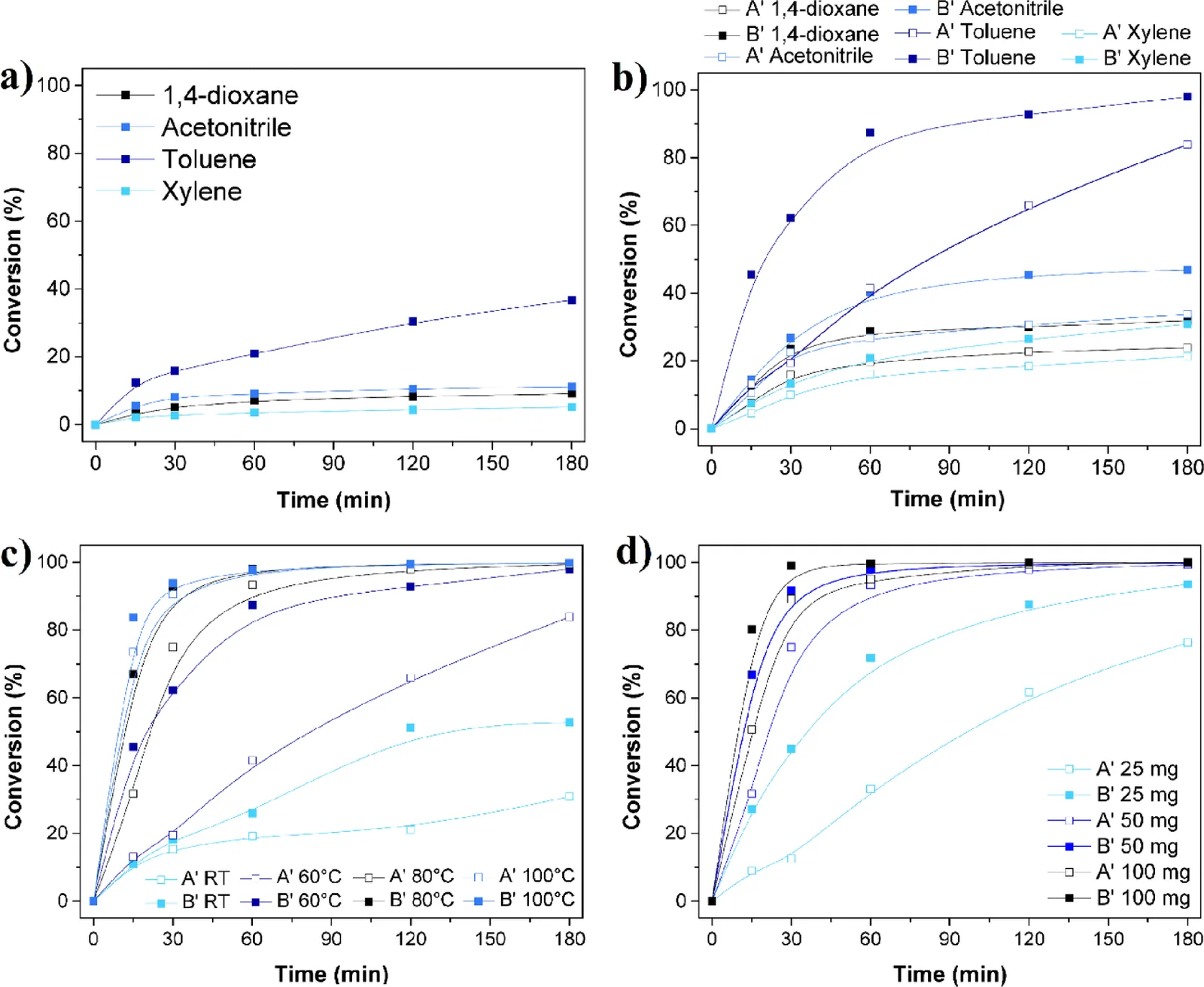
Conversion curves under different conditions for the benzaldehyde–malononitrile reaction: (**a**) effect of solvent on a) **1’** and (**b**) catalysts **A’** and **B’** at 60°C, (**c**) effect of temperature on conversion using **A’** and **B’**, and (**d**) effect of catalyst loading (**A’** and **B’**) at 80 °C.

The effect of solvent on the catalytic performance was investigated for the reaction between benzaldehyde and malononitrile at 60°C using catalysts **1’**, **A’**, and **B’**. The solvents tested included 1,4-dioxane, acetonitrile, toluene, and *o*-xylene, and the corresponding conversions are summarized in Fig. 4a and b. Among the investigated solvents, toluene provided the highest catalytic activity, affording conversions of 37% for **1’** (Fig. 4a), 84% for **A’**, and 98% for **B’** (Fig. 4b) after 180 min. The superior catalytic performance observed in toluene can be rationalized based on solvent–catalyst and solvent–transport effects. More polar solvents such as acetonitrile and 1,4-dioxane contain donor atoms (*N* and *O*, respectively) capable of interacting with coordinatively unsaturated Cu(II) sites, which may lead to partial blocking of CUSs and reduced catalytic efficiency. In contrast, non-coordinating aromatic solvents such as toluene and *o*-xylene minimize competitive coordination at Cu(II) active centers. However, the slightly lower performance observed in *o*-xylene compared to toluene may be associated with the larger molecular size of *o*-xylene, which can introduce steric limitations and reduce diffusion efficiency within the porous framework. Taken together, toluene provides an optimal balance between minimal competitive coordination to active sites and efficient mass transport within the hierarchical pore structure, resulting in superior catalytic performance. The significantly higher catalytic performance observed for materials **A’** and **B’** compared to parent HKUST-1 (**1’**) can also be attributed to improved mass transport properties associated with the presence of additional mesoporous domains. The presence of these larger transport channels facilitates faster diffusion of reactant molecules toward internal active sites and improves the removal of reaction products from the pore network, resulting in higher effective catalytic turnover. In addition to enhanced diffusion, the hierarchical pore architecture likely improves accessibility of Cu(II) CUSs by reducing steric limitations for substrate molecules approaching internal catalytic centers. The slightly higher activity observed for **B’** compared to **A’** is consistent with its higher mesopore contribution, as confirmed by PSD analysis. Subsequent optimization studies were therefore carried out using catalysts **A’** and **B’**. Based on these results, toluene was selected as the optimal reaction medium for further catalytic studies.

The effect of temperature on the Knoevenagel condensation of benzaldehyde with malononitrile in toluene was investigated at four temperatures (RT (room temperature), 60°C, 80°C, and 100°C) using catalysts **A’** and **B’**. As expected, conversion increased with increasing reaction temperature (see Fig. 4c). However, increasing the temperature from 80°C to 100°C resulted in only a marginal improvement in final conversion after 180 min of reaction. For **A’**, conversion increased from 93% at 80°C to 97% at 100°C after 180 min. In contrast, catalyst **B’** exhibited only a minimal increase in conversion, from 98% to 99% under the same conditions. Notably, differences in catalytic activity between **A’** and **B’** were most pronounced during the initial reaction period (0–60 min), where catalyst **B’** consistently exhibited higher reaction rates compared to **A’**. This behavior is attributed to the higher mesopore contribution in sample **B’**, which improves mass transport to internal active sites and is most pronounced during the initial stage of the reaction. These early-stage kinetic differences were subsequently used for calculation of apparent activation energies. Based on these results, 80 °C was selected as the optimal reaction temperature for subsequent catalytic studies using substituted benzaldehyde derivatives, as it provides near-maximal conversion while avoiding unnecessary thermal energy input.

The effect of catalyst loading on the Knoevenagel condensation of benzaldehyde with malononitrile at 80°C in toluene was further investigated using **A’** and **B’** at three different catalyst amounts (25, 50, and 100 mg) (Fig. 4d). Increasing the catalyst amount from 25 mg to 50 mg resulted in a significant increase in conversion for both catalysts, from 75% to 97% for **A’** and from 93% to 99% for **B’** after 180 min of reaction. This effect was more pronounced for catalyst **A’**, where the conversion increased by up to 12%. In contrast, a further increase in catalyst loading to 100 mg resulted in only a negligible improvement in conversion, with nearly complete conversion being achieved after 180 min of reaction. This behavior is consistent with the expected increase in the number of accessible CUSs with increasing catalyst loading, which enhances the probability of reactant adsorption and catalytic turnover. Based on these results, a catalyst loading of 50 mg was selected for subsequent catalytic studies involving substituted benzaldehyde derivatives, as it provides near-maximum catalytic efficiency while maintaining efficient catalyst utilization.

The influence of reaction time on the Knoevenagel condensation of benzaldehyde with malononitrile was evaluated under the optimized solvent, temperature, and catalyst loading conditions (toluene, 80°C, 50 mg catalyst) over a time interval of 0–180 min (Fig. S4 in ESI). The conversion increased rapidly during the initial reaction stage, reaching 93% for **A’** and 98% for **B’** after 60 min. Nearly complete conversion for **A’** and complete conversion for **B’** were achieved after 120 min, with quantitative conversion for both catalysts after 180 min. Although quantitative conversion was reached at longer reaction times, the most significant increase in conversion occurred within the first 60 min. Extending the reaction beyond this time resulted only in marginal improvements, indicating that the reaction approaches completion within the first hour under the applied conditions. Considering the high conversions achieved within a relatively short reaction time, 60 min was selected as the optimal reaction time, as it ensures near-quantitative conversion while maintaining high process efficiency, which is desirable for catalytic applications.

Based on the optimization studies, the final reaction conditions for subsequent catalytic experiments were established as follows: toluene as solvent, reaction temperature of 80°C, catalyst loading of 50 mg, and reaction time of 60 min. Under the optimized reaction conditions, **A’** and **B’** achieved conversions of 93% and 98%, respectively, with complete selectivity toward the desired *α*,*β*-unsaturated nitrile product. These results are comparable or superior to those reported for other MOF-based catalysts under similar or more demanding reaction conditions. For example, UiO-66 exhibited only 3% conversion (100 °C, 50 mg catalyst, 60 min), while Al-MIL-100 reached 30% conversion under identical temperature and catalyst loading conditions^10^. Similarly, Fe-MIL-101-NH^2^ and Al-MIL-101-NH^2^ achieved conversions of 78% and 61%, respectively, at 80 °C using higher catalyst loading (70 mg) and shorter reaction time (30 min)^53^. These comparisons highlight the high catalytic efficiency of the hierarchical HKUST-1 materials developed in this work.

One of the key advantages of heterogeneous catalysis is the facile separation of the catalyst from the reaction medium, together with the possibility of regeneration and reuse. The recyclability of **A’** and **B’** was evaluated in the Knoevenagel condensation of benzaldehyde with malononitrile at 80°C for 60 min in toluene over five consecutive reaction cycles (Fig. S5 in ESI). After each catalytic run, the catalyst was separated by centrifugation, washed with methanol, reactivated at 120°C for 60 min, and reused in the subsequent cycle. Both catalysts exhibited high stability during repeated use. The conversion gradually decreased from 93% to 79% for **A’** and from 98% to 84% for **B’** over five cycles. The gradual decrease in catalytic performance upon recycling can be attributed to partial pore blocking by strongly adsorbed reaction species, minor loss of catalyst during recovery and handling, and possible partial deactivation of surface active sites. Despite this decrease, both catalysts retained high catalytic activity after five cycles, confirming good recyclability and structural robustness under the applied reaction conditions.

The subsequent objective was to investigate the influence of substituents with electron-donating and electron-withdrawing characteristics, as well as the effect of their positional orientation (*ortho* vs *para*), on the catalytic performance (Table 3). Halogen substituents (F, Cl, Br) and the nitro group (NO_2_) were therefore selected as representative substituents covering a broad range of electronic effects. For **B’**, which exhibits a higher contribution of mesopores, the catalytic activity was predominantly governed by electronic effects. For *ortho*-substituted benzaldehydes (Fig. S6a in ESI), the conversion decreased with decreasing electron-withdrawing strength of the substituent, following the order NO_2_ > F > Cl > Br. A similar trend was observed for *para*-substituted derivatives (Fig. S6b in ESI), except for the fluoro substituent, where the lowest conversion was observed. This deviation can be attributed to the positive mesomeric effect of the *para*-fluoro substituent, which partially counteracts its negative inductive effect. The high activity observed for nitro-substituted derivatives can be associated with strong polarization of the carbonyl group induced by the electron-withdrawing NO_2_ substituent, which facilitates nucleophilic attack of the activated methylene compound and stabilizes the corresponding reaction intermediate. In contrast, for catalyst **A’**, the catalytic performance was influenced not only by electronic effects but also by steric factors, which is consistent with the predominantly microporous nature of this material. For *ortho*-substituted benzaldehydes, conversion decreased with increasing substituent size following the order F > Cl > Br > NO_2_. For *para*-substituted derivatives (Fig. S6b in ESI), the order Cl > F > Br > NO_2_ was observed, again reflecting the balance between inductive and mesomeric substituent effects. A more detailed comparison of *para*-substituted benzaldehydes revealed that conversions obtained using **B’** reached 47%, 97%, 95%, and 96% for 4-fluoro-, 4-chloro-, 4-bromo-, and 4-nitrobenzaldehyde, respectively, while **A’** afforded conversions of 31%, 91%, 20%, and 6% under identical reaction conditions. These results demonstrate the beneficial effect of hierarchical porosity on catalytic performance, particularly for substrates affected by steric or diffusion limitations. The catalytic performance of the **A’** and **B’** materials is comparable to that reported for CAU-1-NH_2_ under similar reaction conditions^53^. For example, CAU-1-NH_2_ exhibits conversions of 92%, 90%, 69%, and 82% for 4-fluoro-, 4-chloro-, 4-bromo-, and 4-nitrobenzaldehyde, respectively, demonstrating performance comparable to the hierarchical HKUST-1 catalysts developed in this study.

The influence of the number of substituents was further investigated using di- and trifluoro-substituted benzaldehydes. As shown in Fig. S7 in ESI, the catalytic activity of these derivatives decreased compared to monosubstituted analogues. The observed reactivity order for both catalysts was: 2-fluorobenzaldehyde > 2,4-difluorobenzaldehyde > 4-fluorobenzaldehyde > 2,4,6-trifluorobenzaldehyde. In general, **B’** provided higher conversions than **A’** across the investigated fluorinated substrates, consistent with improved mass transport properties associated with the hierarchical pore structure. For fluorinated benzaldehydes, conversions obtained using **B’** were typically in the range of ~ 45–90%, whereas **A’** afforded conversions in the range of ~ 5–84%, depending on the substitution pattern and degree of fluorination. For example, conversions of ~ 90%, ~ 47%, ~ 51%, and ~ 10% were obtained using **B’** for 2-fluoro-, 4-fluoro-, 2,4-difluoro-, and 2,4,6-trifluorobenzaldehyde, respectively, compared to ~ 84%, ~ 31%, ~ 37%, and ~ 5% for **A’** under identical reaction conditions (80 °C, 60 min, toluene, see Table 3). The gradual decrease in catalytic activity with increasing number of fluorine substituents can be attributed primarily to steric and diffusion-related effects. Introduction of multiple fluorine substituents increases the effective molecular size and steric hindrance around the carbonyl group, which can limit substrate accessibility to internal Cu(II) CUSs, particularly within microporous regions of the framework. In addition, strong cumulative electron-withdrawing inductive effects of multiple fluorine substituents can modify adsorption strength and electronic activation of the carbonyl group, potentially leading to less favorable catalyst–substrate interactions. The relatively high activity observed for 2-fluorobenzaldehyde compared to 4-fluorobenzaldehyde is consistent with the strong inductive activation of the carbonyl group in the *ortho* position, while the *para*-fluoro substituent exhibits partial positive mesomeric contribution, reducing the overall electrophilicity of the carbonyl carbon.

Finally, the influence of alkyl substituents with different steric demands and substitution patterns was investigated using mono-substituted benzaldehydes in the *para* position and disubstituted benzaldehydes in the *meta* positions (Fig. S8 in ESI). This substrate set enabled evaluation of both increasing steric bulk of the alkyl substituent (methyl < isopropyl < *tert-*butyl) and the effect of multiple substituents positioned close to the reactive carbonyl group. In this case, **B’** again exhibited higher catalytic activity than **A’**, however, the overall activity for alkyl-substituted substrates was significantly lower compared to substrates bearing electron-withdrawing substituents. For alkyl-substituted benzaldehydes, conversions obtained using **B’** were typically in the range of ~ 6–16%, whereas **A’** afforded conversions in the range of ~ 5–11%, depending on substituent size and substitution pattern. For example, conversions of approximately ~ 16%, ~ 14%, ~ 6%, and ~ 7.5% were obtained using **B’** for 4-methyl-, 3,5-dimethyl-, 4-isopropyl-, and 4-*tert*-butylbenzaldehyde, respectively, compared to ~ 11%, ~ 10%, ~ 5%, and ~ 6.5% for **A’** under identical reaction conditions. No detectable catalytic activity was observed for either catalyst in the case of 3,5-di-*tert*-butylbenzaldehyde. The observed reactivity trend (4-methylbenzaldehyde > 3,5-dimethylbenzaldehyde > 4-*tert*-butylbenzaldehyde > 4-isopropylbenzaldehyde > 3,5-di-*tert*-butylbenzaldehyde) indicates that steric effects dominate over electronic effects for these substrates. This behavior can be rationalized by considering the relationship between substrate size and pore architecture. Increasing alkyl substitution leads to an increase in effective molecular diameter, progressing from approximately ~ 0.70 nm for methyl-substituted benzaldehydes to ~ 0.85–0.95 nm for isopropyl derivatives and approaching ~ 1.0–1.1 nm for *tert*-butyl-substituted substrates. In the case of 3,5-di-*tert*-butylbenzaldehyde, the effective molecular size can exceed ~ 1.2 nm^54^. As the substrate size approaches the characteristic dimensions of the HKUST-1 framework, molecular diffusion becomes increasingly hindered. Although the dimensions of bulky alkyl-substituted benzaldehydes may be comparable to the larger cages (~ 0.9–1.1 nm), their transport through the structure is governed primarily by the significantly narrower pore window apertures (~ 0.35–0.45 nm). Consequently, diffusion between adjacent cages can be strongly restricted, limiting access to internal Cu(II) CUS active sites and leading to the observed decrease in catalytic performance for sterically demanding substrates. These results collectively indicate that catalytic performance in HKUST-1 is governed by a balance between intrinsic active site density and substrate transport limitations, with window-mediated diffusion becoming dominant for bulky molecules.

**Table 3:** Results of the Knoevenagel condensation of aldehydes with malononitrile at 80°C after 60 min using catalysts **A’** and **B’**.

Entry	Aldehyde	Product	HKUST-1	Conversion (%)
**1**			**A’**	**93**
			**B’**	**98**
**2**			**A’**	**84**
			**B’**	**90**
**3**			**A’**	**40**
			**B’**	**82**
**4**			**A’**	**48**
			**B’**	**85**
**5**			**A’**	**36**
			**B’**	**96**
**6**			**A’**	**31**
			**B’**	**47**
**7**			**A’**	**91**
			**B’**	**97**
**8**			**A’**	**20**
			**B’**	**95**
**9**			**A’**	**6**
			**B’**	**96**
**10**			**A’**	**37**
			**B’**	**51**
**11**			**A’**	**6**
			**B’**	**9**
**12**			**A’**	**11**
			**B’**	**16**
**13**			**A’**	**10**
			**B’**	**14**
**14**			**A’**	**5**
			**B’**	**6**
**15**			**A’**	**7**
			**B’**	**8**
**16**			**A’**	**0**
			**B’**	**0**

To verify the heterogeneous nature of the catalytic process and to exclude the contribution of leached active species, a hot filtration leaching test was performed using benzaldehyde and malononitrile at 80°C. After 15 min of reaction, the catalyst was removed from the reaction mixture by filtration, and the filtrate was allowed to react for an additional 45 min under identical reaction conditions. Aliquots were collected at 15 min intervals and analyzed by GC. As shown in Fig. S9 in ESI, no further increase in conversion was observed after catalyst removal, with conversions remaining constant at 36% for **A’** and 66% for **B’**. AAS analysis of the post-reaction solution confirmed that no Cu(II) ions were detected in the liquid phase. These results indicate the absence of catalytically active Cu species in the solution and confirm that both materials operate as stable heterogeneous catalysts under the applied reaction conditions.

### Activation energy evaluation

The evaluation of the activation energy for the catalytic reaction between benzaldehyde and malononitrile was carried out as follows. A catalytic mechanism was considered, in which benzaldehyde is bound to the surface of the catalyst, specifically to the available coordination unsaturated sites (CUSs) of the Cu(II) metal centers in this case. Malononitrile is supplied from the solution phase, while the reaction proceeds at the Cu(II) sites. The resulting product is subsequently desorbed back into the solution, with only trace amounts retained within the framework (see TG analysis below). Based on this assumption, the Eley-Rideal kinetic model (1), which describes this type of catalytic mechanism, was applied.

1 $$\:r=\:\frac{k{K}_{A}{C}_{A}{C}_{B}}{1+{K}_{A}{C}_{A}}$$

Where *r* is the reaction rate (mol L^-1^ min^-1^, *k* is the rate constant of the surface reaction (min^-1^, *K*_*a*_ is the adsorption constant of benzaldehyde (L mol^-1^, *C*_*a*_ is the concentration of benzaldehyde (mol L^-1^, and *Cβ* is the concentration of malononitrile (mol L^-1^.

Since the reaction proceeds at active sites where benzaldehyde is adsorbed and activated, and its activated form participates directly in the reaction, adsorption measurements were carried out at boundary temperatures (RT and 100°C) to determine the limiting surface coverage of active sites by benzaldehyde. Unfortunately, adsorption isotherms corresponding to type *III* were obtained for both materials **A’** and **B’**, from which the surface coverage could not be directly determined. Therefore, an approach based on the normalization of benzaldehyde uptake to the number of Cu(II) ions was applied, with the calculation referenced to 1 g of HKUST-1. The surface coverage of active sites was expressed as follows:

2 $$\:{\theta\:}_{Cu}=\:\frac{{n}_{BA}}{{n}_{Cu}}=\frac{{Q}_{e}/{M}_{BA}}{{n}_{Cu}}$$

Where *Q*_*e*_ is the adsorbed amount (g g^− 1^), *M*_*BA*_ is the molar mass (g mol^− 1^) of benzaldehyde, and *n*_*Cu*_ is the amount (mol) of copper in HKUST-1. The values of *C*_*0*_ versus *θ* are presented in Fig. S10 in ESI. Based on the obtained data for material **A’** (*θ* = 2.39 at RT and *θ* = 1.40 at 100°C) and material **B’** (*θ* = 3.07 at RT and *θ* = 1.83 at 100 °C), it can be concluded that all CUSs of Cu(II) ions are occupied. The excess benzaldehyde molecules are physisorbed within the pores. Based on this assumption, and considering the condition *θ* = 1, the Eley–Rideal rate expression can be simplified to the following final form:

3 $$\:r=k{\theta\:}_{A}{C}_{B}$$

For the fitting of the kinetic data, the following form of the equation was used:

4 $$\:{C}_{A}\left(t\right)={C}_{A}0\mathrm{e}\mathrm{x}\mathrm{p}(-kt)$$

This indicates pseudo-first-order behavior. Considering the nature of the reaction between malononitrile and benzaldehyde, the reaction would be expected to follow pseudo-second-order kinetics, however, due to the saturated surface of the catalyst, the observed behavior deviates from this assumption. The resulting rate constants *k* and *R*^*2*^ values are summarized in Table 4. As can be seen, the rate constant increases with increasing temperature (from RT to 100°C), rising from 0.0017 to 0.0855 min^− 1^ for sample **A’**. The **B’** sample exhibited faster catalytic activity, with *k* values ranging from 0.0104 to 0.1159 min^− 1^. These values were subsequently used for the calculation of the activation energy.

**Table 4:** Temperature dependence of the effective rate constant.

Temp.	Sample	k (min^− 1^)	*R* ^2^
RT	**A’**	0.0017 ± 3.8 × 10^− 4^	0.8047
	**B’**	0.0104 ± 5.4 × 10^− 4^	0.9515
60 °C	**A’**	0.0093 ± 4.7 × 10^− 4^	0.99937
	**B’**	0.0342 ± 2.9 × 10^− 4^	0.9885
80 °C	**A’**	0,0.0386 ± 5.5 × 10^− 3^	0.9725
	**B’**	0.0756 ± 2.2 × 10^− 3^	0.9990
100 °C	**A’**	0.0855 ± 3.2 × 10^− 3^	0.9986
	**B’**	0.1159 ± 7.4 × 10^− 3^	0.9972

**Fig. 5:**
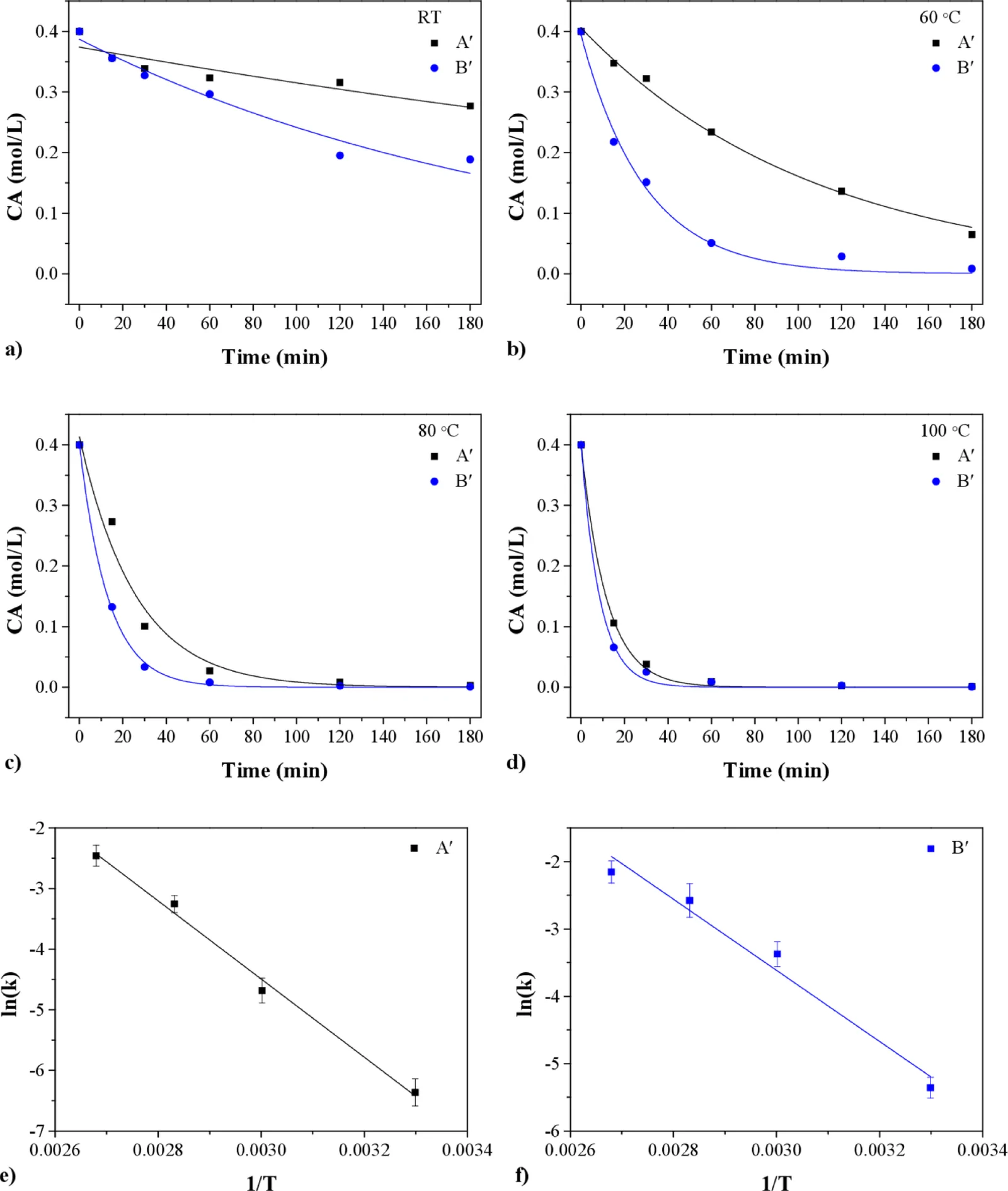
Second-order fits of kinetics data during the catalyzed reaction of benzaldehyde concentration with malononitrile over **A’** and **B’** catalysts at (**a**) room temperature (RT), (**b**) 60°C, (**c**) 80°C, and (**d**) 100°C; symbols represent experimental data, while solid lines correspond to non-linear regression according to second-order kinetics. Arrhenius plots of ln(*K*_eff_) versus 1/*T* for the catalyzed reaction of benzaldehyde with malononitrile over (**e**) **A’** and (**f**) **B’** catalysts. Solid lines represent linear regression fits used to determine the effective activation energy.

The activation energy was determined from the Arrhenius plot of ln(*k*) versus 1/*T* (see Fig. 5), where the slope of the linear regression corresponds to −*E*_a,eff_/*R*^55^. Linear regression of the plot yielded an effective activation energy of *E*_a,eff_ = 53.6 ± 3.1 kJ mol^-1^ for **A’**, whereas a lower value of *E*_a,eff_ = 43.9 ± 5.2 kJ mol^-1^ was obtained for **B’**. Comparable activation energy ranges have also been reported for related MOF-based catalytic systems in the literature^56^. The obtained activation energy values represent a combined energetic contribution of the surface reaction and benzaldehyde adsorption, which is consistent with the simplified Eley–Rideal mechanism to pseudo-second order, for which the kinetic constant was defined as *k* = *kK*_a_. The lower *E*ₐ value observed for **B’** indicates a more energetically favorable course of the elementary reaction steps and correlates with the higher *k* values observed over the entire investigated temperature range. To verify that the calculated kinetic parameters are not affected by internal diffusion limitations, the Weisz–Prater criterion was evaluated based on Eq. (5).

5 $$\:{C}_{WP}=\frac{{r}_{obs}{\rho\:}_{p}{R}_{p}^{2}}{{C}_{s}{D}_{eff}}$$

Where *C*_*wp*_ is the Weisz–Prater number, *r*_*obs*_ is the experimental reaction rate (mol kg^-1^ s^-1^, *ρ*_*p*_ is the catalyst density (kg m^-3^, *R*_*p*_ is the particle radius (m), *C*_*s*_ is the reactant concentration at the surface (mol m^-3^, and *D*_*eff*_ is the effective diffusion coefficient (m^2^ s^-1^.

The individual parameters were obtained as follows: the value of *r*_*obs*_ was calculated from experimental measurements at different temperatures (**A’**: RT to 100°C; *r*_*obs*_ = 0.00969, 0.01164, 0.02818, and 0.06533 mol kg^-1^ s^-1^; **B’**: RT to 100°C; *r*_*obs*_ = 0.00987, 0.04053, 0.05947, and 0.07431 mol kg^-1^ s^-1^. The catalyst density was determined using a pycnometric method, yielding a value of *ρ*_*p*_ = 880 kg m^-3^ for both materials **A’** and **B’**. The particle radius was calculated from experimentally determined particle size data obtained by DLS measurements, giving *R*_*p*_ = 8.72 × 10^− 7^ m for **A’** and *R*_*p*_ = 7.78 × 10^− 7^ m for **B’**. The value of *C*_*s*_ was taken as 400 mol m^-3^, assuming a well-mixed system with a powdered catalyst. The parameter *D*_*eff*_ for benzaldehyde (1.08 × 10^–10^ m^2^ s^-1^ was adopted from the literature^57,58^. The calculated Weisz–Prater values for material **A’** ranged from 1.50 × 10^− 4^ at RT, through 1.80 × 10^− 4^ at 60 °C and 4.36 × 10^− 4^ at 80 °C, to 1.01 × 10^− 3^ at 100 °C. For material **B’**, the corresponding values were 1.22 × 10^− 4^, 4.99 × 10^− 4^, 7.32 × 10^− 4^, and 9.15 × 10^− 4^ at RT, 60, 80 and 100 °C, respectively. All values were significantly lower than 0.1, indicating negligible intraparticle diffusion limitations.

### Characterization of catalysts after catalytic reactions

**Fig. 6:**
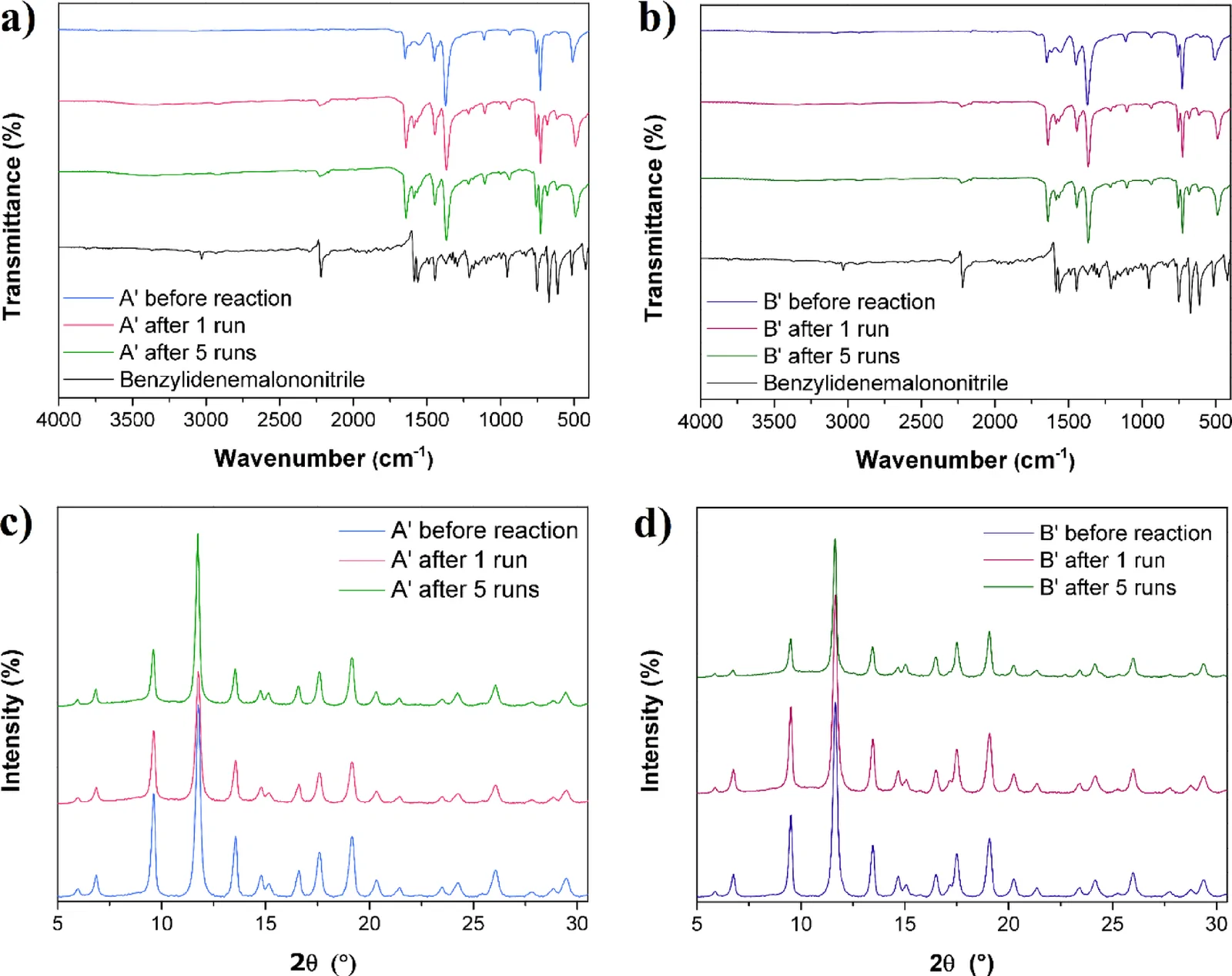
Comparison of the FT-IR spectra of catalysts (**a**) **A’** and (**b**) **B’** recorded before, after one and five catalytic runs, together with the spectrum of the reaction product (benzylidenemalononitrile). Comparison of the PXRD patterns of catalysts (**c**) **A’** and (**d**) **B’** recorded before catalysis, after one and five catalytic runs.

The potential immobilization of reaction product or reactants within the catalyst framework was investigated by FT-IR spectroscopy. Figure 6a shows the FT-IR spectra of benzylidenemalononitrile and catalyst **A’** recorded before catalysis and after one catalytic run (80°C, 60 min, toluene). The only notable difference between the spectra is the appearance of additional absorption bands at 2225, 1586, 679, and 614 cm^− 1^. These bands correspond to product molecules confined within the pores and can be assigned to the stretching vibration *ν*(C ≡ N) (2225 cm^− 1^), the conjugated *ν*(C = C) vibration (1586 cm^− 1^), and lower-frequency vibrations at 679 and 614 cm^− 1^ associated with aromatic *γ*(C–H) out-of-plane bending and bending *δ*(C ≡ N) vibration^59^. The absence of absorption bands corresponding to *ν*(CH_2_) (malononitrile) and *ν*(C = O) (benzaldehyde) vibrations confirms that the immobilized species corresponds to a reaction product rather than unreacted substrates. The FT-IR spectrum of **A’** after the fifth catalytic cycle shows no significant changes compared to the spectrum after the first run, indicating that the material retains its structural integrity upon repeated catalytic use. As evident from Fig. 6b, catalyst **B’** exhibits analogous spectral behavior. The structural stability of the catalysts after catalytic testing was further evaluated by PXRD. As shown in Fig. 6c and d, no significant differences were observed in the diffraction patterns of **A’** and **B’** before and after catalysis, confirming preservation of the original crystal structure and crystallinity. The structural stability of both materials was additionally verified after the fifth catalytic cycle, where the PXRD patterns again showed no detectable structural changes. Furthermore, the stability of **A’** and **B’** was examined after catalytic reactions involving substituted benzaldehydes. The corresponding PXRD patterns (Fig. S11 in ESI) again confirmed preservation of structural integrity and crystallinity under the applied reaction conditions. Thermogravimetric analysis was further performed to evaluate the thermal stability of the catalysts before and after catalytic cycling and to estimate the amount of retained guest species within the framework (Fig. S12 in ESI). Since the samples contain different amounts of adsorbed solvents or guest molecules within the porous structure, the thermoanalytical curves were normalized at 250°C similarly to previous reports^60,61^. The mass loss associated with the removal of physically adsorbed species was thus redistributed between the decomposition step of the organic framework and the final residual mass. The TG curves of the **A** and **B** materials exhibit an initial low-temperature mass loss below approximately 250°C corresponding to the release of residual solvent molecules confined within the porous structure. The frameworks remain thermally stable up to approximately 300°C, followed by a major decomposition step in the temperature range of approximately 300–360°C associated with decomposition of the BTC linker and collapse of the HKUST-1 framework. The final residual mass above 400°C corresponds to CuO formed during oxidation of the organic framework under air atmosphere. The normalized mass losses (Fig. S12b in ESI) attributed to the organic fraction were 59.59% for **A** and 60.60% for **B**, while the residual masses were 40.41% and 39.40%, respectively, which are in good agreement with the expected composition of HKUST-1-derived CuO residue (clcd residual mass 39.45%). The original non-normalized TG curves of the catalysts after five catalytic cycles are shown in Fig. S12a in ESI. In contrast to the FT-IR spectra, no distinct additional decomposition step corresponding exclusively to retained benzylidenemalononitrile was observed after catalysis. Instead, the decomposition associated with the retained guest molecules likely overlaps with the main framework decomposition step, suggesting relatively strong interactions between the confined product molecules and the porous framework^62^. After normalization, the mass losses corresponding to the organic fraction increased to 65.03% for **A’** and 67.95% for **B’** after five catalytic cycles. By subtracting the framework-related organic contribution of the fresh catalysts, the amount of retained benzylidenemalononitrile confined within the pores was estimated to be 5.44 wt.% (54.4 mg g^− 1^, 0.353 mmol g^− 1^) for **A’** and 7.35 wt% (73.5 mg g^− 1^, 0.477 mmol g^− 1^) for **B’**. These results confirm partial pore occupation by retained reaction products after repeated catalytic use, which is consistent with the FT-IR results, while PXRD analysis demonstrates that the overall framework structure and crystallinity remain preserved.

## Conclusion

In this work, hierarchically porous HKUST-1 materials (**A** and **B**) were successfully synthesized via a cooperative template-directed strategy, enabling controlled introduction of mesoporosity while preserving the characteristic framework. Structural, spectroscopic, and textural characterization confirmed retention of the intrinsic HKUST-1 crystal structure together with high specific surface areas of 1787, 1554, and 1690 m^2^ g^− 1^ for activated samples **1’**, **A’**, and **B’**, respectively. The hierarchical materials exhibited development of mesoporous domains with increased mesopore volume to 0.159 cm^3^ g^− 1^ for **A’** and 0.210 cm^3^ g^− 1^ for **B’**, confirming successful modification of the pore architecture.

The catalytic performance of the activated materials was evaluated in the Knoevenagel condensation of benzaldehyde derivatives with malononitrile. Under optimized conditions (toluene, 80°C, 50 mg catalyst, 60 min), conversions of 93% and 98% were achieved for **A’** and **B’**, respectively. The hierarchical catalysts demonstrated significantly improved catalytic activity compared to the parent microporous material, which can be attributed to enhanced mass transport and improved accessibility of internal Cu(II) CUSs. Notably, the catalytic trends suggest that total specific surface area alone does not control the observed activity. Rather, mesopore volume and the resulting improvement in diffusion pathways represent the key parameters, which is supported by the correlation between nitrogen physisorption results and catalytic performance. Kinetic analysis based on the Eley–Rideal model confirmed higher effective kinetic constants (*K*_*eff*_) for **B’** over the entire investigated temperature range. Arrhenius analysis revealed an effective *E*_*a*_ of 53.6 ± 3.1 kJ mol^− 1^ for **A’** and 43.9 ± 5.2 kJ mol^− 1^ for **B’**. The lower activation energy observed for **B’** indicates a more energetically favorable reaction pathway, which can be attributed to improved diffusion efficiency and enhanced accessibility of internal active sites enabled by the pore structure.

Systematic catalytic studies revealed strong correlations between substrate structure, pore architecture, and catalytic performance. High conversions were obtained for substrates bearing electron-withdrawing substituents (up to ~ 96–98% conversion for nitro- and halogen-substituted benzaldehydes). In contrast, sterically demanding alkyl-substituted substrates exhibited significantly lower conversions (~ 6–16% for **B’** and ~ 5–11% for **A’**), confirming that molecular size and diffusion limitations play a dominant role for bulky substrates. These findings demonstrate that cooperative templating enables tuning of transport properties without compromising the intrinsic catalytic functionality of the HKUST-1 framework.

Hot filtration experiments together with AAS analysis confirmed the absence of detectable Cu(II) leaching, demonstrating the truly heterogeneous nature of the catalytic process. Furthermore, recyclability experiments showed only a moderate decrease in catalytic activity after five cycles, with conversions remaining at approximately 76% for **A’** and 83% for **B’**. Post-catalysis FT-IR, PXRD, and TG analyses confirmed structural stability and preservation of crystallinity of the HKUST-1 catalysts under repeated catalytic operation and under various substrate conditions.

Overall, the catalytic performance of the prepared hierarchical HKUST-1 materials is comparable to or superior to several previously reported MOF-based catalysts under similar reaction conditions, highlighting the effectiveness of hierarchical pore engineering for improving catalytic efficiency in MOF systems. The results presented in this study demonstrate that cooperative template-directed synthesis represents a promising strategy for rational design of hierarchical MOF catalysts with optimized transport properties. Future research may focus on further tuning of mesopore connectivity and distribution to maximize accessibility of internal active sites. In addition, extension of this strategy to other MOF systems and catalytic reactions involving larger or more complex molecules may further expand the applicability of hierarchical MOFs in heterogeneous catalysis. Integration of hierarchical pore engineering with post-synthetic functionalization or incorporation of redox-active sites represents a promising direction toward the development of next-generation multifunctional MOF catalysts.

## Supplementary Information

Below is the link to the electronic supplementary material.

Supplementary Material 1

## Data Availability

The datasets used and/or analysed during the current study available from the corresponding author on reasonable request.
