# Supplementary material for: Effect of porosity on catalytic performance of HKUST-1 in Knoevenagel condensation
Source: Sci Rep. 2026 Jun 23;16:28658. doi: 10.1038/s41598-026-59395-w (PMC13575166; doi:10.1038/s41598-026-59395-w)
Supplement: Supplementary file 1 [file 41598_2026_59395_MOESM1_ESM.docx]

**Electronic Supplementary Information**

**(ESI)**

**Effect of Porosity on Catalytic Performance of HKUST-1 in Knoevenagel Condensation**

Nikola Vargová^1^, Milica Želinská^1^, Ľuboš Zauška^1^, Lucie Zelená^1,2^, Rastislav Serbin^3^, Daria Striežovská^4^, Jozef Bednarčík^4^, Tomáš Zelenka^2^, Marta Férová^2^, Peter Obšatník^1^, Vladimír Zeleňák^1^, Miroslav Almáši^1^*, Nikolas Király^1^

1 *Department of Inorganic Chemistry, Institute of Chemistry, Faculty of Science, Pavol Jozef Šafárik University in Košice, Moyzesova 11, SK-041 54 Košice, Slovakia*
2 *Department of Chemistry, Faculty of Science, University of Ostrava, 30. Dubna 22, CZ-702 00 Ostrava, Czech Republic*
3 *Department of Analytical Chemistry, Institute of Chemistry, Faculty of Science, Pavol Jozef Šafárik University in Košice, Moyzesova 11, SK-041 54 Košice, Slovakia*
4 *Department of Condensed Matter Physics, Institute of Physics, Faculty of Science, Pavol Jozef Šafárik University in Košice, Moyzesova 11, SK-041 54 Košice, Slovakia*

*Corresponding author:* [*miroslav.almasi@upjs.sk*](mailto:miroslav.almasi@upjs.sk)

| **Table of Contents** | **Page** |
| --- | --- |
| **Fig. S1** IR spectra of **1**, **A** and **B**. | S3 |
| **Fig. S2** IR spectra of **1', A'** and **B'** after activation process | S3 |
| **Fig. S3** NLDFT fitting of the nitrogen adsorption isotherm in a semi-logarithmic scale: a) full-range fit (10^-6^–0.995 p/p_0_) with poor agreement at low pressures (1.84% error); b) adjusted fit for p/p_0_ ≳ 0.01 with improved agreement (0.15% error) | S4 |
| **Fig. S4** Conversion curves for the reaction between benzaldehyde and malononitrile at 80 °C using catalysts **A'** and **B'** over a reaction time of up to 180 minutes | S5 |
| **Fig. S5** Conversions of reused catalysts **A'** and **B'** for reaction of benzaldehyde and malononitrile at 80°C. | S5 |
| **Fig. S6** Comparison of conversion for reaction of a) ortho- and b) para-substitueted benzaldehydes with malononitrile at 80 °C using **A'** and **B'** as catalysts | S6 |
| **Fig. S7** Comparison of conversion for reaction of fluoro-substitueted aldehydes at 80 °C using **A'** and **B'** as catalysts | S6 |
| **Fig. S8** Comparison of conversion for reactions of alkyl-substitueted aldehydes with malononitrile at 80 °C using **A'** and **B'** as catalysts | S6 |
| **Fig. S9** Hot filtration leaching test of reaction of benzaldehyde and malononitrile at 80 °C using **A'** and **B'** as catalysts | S7 |
| **Fig. S10** Surface coverage (θ) as a function of initial benzaldehyde concentration (C₀) for materials **A'** a) and **B'** b) at RT and 100 °C. | S7 |
| **Fig. S11** Comparison of the PXRD patterns of catalysts a) **A'** and b) **B'** recorded after reaction of substitueted benzaldehydes and malononitrile at 80°C | S8 |
| **Fig. S12** Comparison of the TG curves of the catalysts before and after five catalytic cycles recorded in the temperature range of 30–900 °C under an air atmosphere: a) original and b) normalized TG curves | S8 |
| **General process for purifying the Knoevenagel condensation products** | S9 |
| **Chemical shifts of prepared compounds** | S9-S12 |
| **References** | S12-S13 |
| **^1^H and ^13^C NMR spectra of prepared compounds** | S14 |
| ^1^H and ^13^C NMR spectra of 2-(2-Fluorobenzylidene)malononitrile^1^(**3b**^1^) | S14 |
| ^1^H and ^13^C NMR spectra of 2-(2-Chlorobenzylidene)malononitrile^2^(**3c**^2^) | S15 |
| ^1^H and ^13^C NMR spectra of 2-(2-Bromobenzylidene)malononitrile^1^(**3d**^1^) | S16 |
| ^1^H and ^13^C NMR spectra of 2-(2-Nitrobenzylidene)malononitrile^1^(**3e**^1^) | S17 |
| ^1^H and ^13^C NMR spectra of 2-(4-Fluorobenzylidene)malononitrile^1^ (**3f**^1^) | S18 |
| ^1^H and ^13^C NMR spectra of 2-(4-Chlorobenzylidene)malononitrile^1^ (**3g**^1^) | S19 |
| ^1^H and ^13^C NMR spectra of 2-(4-Bromobenzylidene)malononitrile^1^ (**3h**^1^) | S20 |
| ^1^H and ^13^C NMR spectra of 2-(4-Nitrobenzylidene)malononitrile^1^ (**3i**^1^) | S21 |
| ^1^H and ^13^C NMR spectra of 2-(2,4-difluorobenzylidene)malononitrile (**3j**) | S22 |
| ^1^H and ^13^C NMR spectra of 2-(2,4,6-trifluorobenzylidene)malononitrile (**3k**) | S23 |
| ^1^H and ^13^C NMR spectra of 2-(4-methylbenzylidene)malononitrile^3^**3l**^3^ | S24 |
| ^1^H and ^13^C NMR spectra of 2-(3,5-dimethylbenzylidene)malononitrile^4^**3m**^4^ | S25 |
| ^1^H and ^13^C NMR spectra of 2-(4-isopropylbenzylidene)malononitrile^1^ **3n**^1^ | S26 |
| ^1^H and ^13^C NMR spectra of 2-(4-tertbutylbenzylidene)malononitrile^5^ **3o**^5^ | S27 |

**Fig. S1** IR spectra of **1**, **A** and **B**.

**Fig. S2** IR spectra of **1**', **A'** and **B'** after activation process.

**Fig. S3** NLDFT fitting of the nitrogen adsorption isotherm in a semi-logarithmic scale: a) full-range fit (10^-6^–0.995 p/p₀) with poor agreement at low pressures (1.84% error); b) adjusted fit for p/p₀ ≳ 0.01 with improved agreement (0.15% error).

**Fig. S4** Conversion curves for the reaction between benzaldehyde and malononitrile at 80 °C using catalysts **A'** and **B**' over a reaction time of up to 180 minutes.

**Fig. S5** Conversions of reused catalysts **A'** and **B'** for reaction of benzaldehyde and malononitrile at 80°C.

**Fig. S6** Comparison of conversion for reaction of a) ortho- and b) para-substitueted benzaldehydes with malononitrile at 80 °C using **A'** and **B'** as catalysts.

**Fig. S7** Comparison of conversion for the reaction of fluoro-substitueted benzaldehydes at 80 °C using **A'** and **B'** as catalysts.

**Fig. S8** Comparison of conversion for reactions of alkyl-substitueted benzaldehydes with malononitrile at 80 °C using **A'** and **B'** as catalysts.

**Fig. S9** Hot filtration leaching test of reaction of benzaldehyde and malononitrile at 80 °C using **A'** and **B'** as catalysts.

**Fig. S10** Surface coverage (*θ*) as a function of initial benzaldehyde concentration (C₀) for materials a) **A'** and b) **B'** at RT and 100 °C.

**Fig. S11** Comparison of the PXRD patterns of catalysts a) **A'** and b) **B'** recorded after reaction of substitueted benzaldehydes and malononitrile at 80°C.

**Fig. S12** Comparison of the TG curves of the catalysts before and after five catalytic cycles recorded in the temperature range of 30–900 °C under an air atmosphere: a) original and b) normalized TG curves.

**General process for purifying the Knoevenagel condensation products**

After completion of the catalytic reaction and separation of the catalyst by centrifugation, the collected reaction supernatant was concentrated under reduced pressure using a rotary evaporator. In most cases, the crude products were purified by repeated recrystallization from absolute ethanol at −4 °C to afford the corresponding pure compounds. The isolated crude products **3k**, **3n**^1^, **3o**^5^ were purified with column chromatography through a short column of silica gel (*n*-hexane/ethyl acetate, 10:1). All the purified products were dried under vacuum on a membrane vacuum pump befor further characterisation. The structures of the products were confirmed by NMR spectroscopy. Most of the synthesized compounds are known, and their NMR spectroscopic data are consistent with those reported in the literature. Structures of the newly prepared compounds **3j** and **3k** were fully characterised and confirmed using 1D and 2D NMR spectra. The NMR spectra of **3j** and **3k** clearly showed all expected structural features, including the characteristic singlet of the newly formed benzylidene (=CH–) proton in the downfield region of the ^1^H NMR spectra, typical signals corresponding to the aromatic fluorinated rings, and carbon resonances attributable to the olefinic carbon and nitrile groups in the ^13^C NMR spectra. The observed chemical shifts and coupling patterns are fully consistent with the proposed structures.

**Chemical shifts of prepared compounds**

**2-(2-Fluorobenzylidene)malononitrile 3b**^1^

 White crystals; Yield; mp ^o^C; **^1^H NMR** (400 MHz, CDCl_3_) *δ*: 8.27−8.31 (m, 1H), 8.10 (s, 1H), 7.62−7.68 (m,1H), 7.32−7.37 (m, 1H), 7.21−7.26 (m, 1H); **^13^C NMR** (100 MHz, CDCl_3_) *δ*: 161.3 (d, *J* = 259.2 Hz), 151.3 (d, *J* = 8.1 Hz), 136.7 (d, *J* = 9.6 Hz), 128.5, 125.3 (d, *J* = 3.8 Hz), 119.3 (d, *J* = 10.4 Hz), 116.5 (d, *J* = 21.5 Hz), 113.3, 112.2, 84.5. These data are consistent with those previously reported in the literature for this compound.^1^

**2-(2-Chlorobenzylidene)malononitrile 3c**^2^

White crystals; Yield; mp ^o^C; **^1^H NMR** (400 MHz, CDCl_3_) *δ*: 8.27−8.28 (m, 1H), 8.17−8.20 (s, 1H), 7.53−7.58 (m, 2H), 7.42−7.49 (m, 1H); **^13^C NMR** (100 MHz, CDCl_3_) *δ*: 156.0, 136.3, 135.0, 130.6, 129.4, 129.0, 127.7, 113.2, 111.9, 85.7. These data are consistent with those previously reported in the literature for this compound.^2^

**2-(2-Bromobenzylidene)malononitrile** **3d**^1^

White crystals; Yield; mp ^o^C; **^1^H NMR** (400 MHz, CDCl_3_) *δ*: 8.22 (s,1H), 8.11−8.12 (m, 1H), 7.74−7.76 (m, 1H), 7.44−7.52 (m, 2H); **^13^C NMR** (100 MHz, CDCl_3_) *δ*: 158.7, 134.9, 133.9, 130.7, 129.7, 128.3, 126.4, 113.0, 111.7, 85.9. These data are consistent with those previously reported in the literature for this compound.^1^

**2-(2-Nitrobenzylidene)malononitrile 3e**^1^

 White crystals; Yield; mp ^o^C; **^1^H NMR** (400 MHz, CDCl_3_) *δ*: 8.45 (s, 1H), 8.36−8.37 (m, 1H), 7.79−7.90 (m, 3H); **^13^C NMR** (100 MHz, CDCl_3_) *δ*: 158.8, 146.7, 134.9, 133.4, 130.4, 126.6, 125.8, 112.2, 110.9, 88.5. These data are consistent with those previously reported in the literature for this compound.^1^

**2-(4-Fluorobenzylidene)malononitrile 3f**^1^

White crystals; Yield; mp ^o^C; **^1^H NMR** (400 MHz, CDCl_3_) *δ*: 7.94−7.99 (m, 2H), 7.76 (s, 1H), 7.21−7.27 (m, 2 H); **^13^C NMR** (100 MHz, CDCl_3_) *δ*: 166.1 (d, *J* = 260.3 Hz), 158.3 (d, *J* = 1.1 Hz), 133.4 (d, *J* = 9.6 Hz), 127.3 (d, *J* = 3.1 Hz), 117.2 (d, *J* = 22.3 Hz), 113.5, 112.5, 82.3 (d, *J* = 2.7 Hz). These data are consistent with those previously reported in the literature for this compound.^1^

**2-(4-Chlorobenzylidene)malononitrile 3g**^1^

 White crystals; Yield; mp ^o^C; **^1^H NMR** (400 MHz, CDCl_3_) *δ*: 7.85−7.88 (m, 2H), 7.75 (s, 1H), 7.51−7.54 (m, 2H); **^13^C NMR** (100 MHz, CDCl_3_) *δ*: 158.3, 141.1, 131.8, 130.0, 129.2, 113.4, 112.3, 83.3. These data are consistent with those previously reported in the literature for this compound.^1^

**2-(4-Bromobenzylidene)malononitrile 3h**^1^

White crystals; Yield; mp ^o^C **^1^H NMR** (400 MHz, CDCl_3_) *δ*: 7.76−7.80 (m, 2H), 7.73 (s, 1H), 7.67−7.71 (m, 2H); **^13^C NMR** (100 MHz, CDCl_3_) *δ*: 158.5, 133.1, 131.8, 129.9, 129.6, 113.5, 112.4, 83.4. These data are consistent with those previously reported in the literature for this compound.^1^

**2-(4-Nitrobenzylidene)malononitrile 3i**^1^

 Yellow crystals; Yield; mp ^o^C **^1^H NMR** (400 MHz, CDCl_3_) *δ*: 8.38−8.41 (m, 2H), 8.07−8.10 (m, 2H), 7.90 (s, 1H); **^13^C NMR** (100 MHz, CDCl_3_) *δ*: 156.9, 150.3, 135.8, 131.3, 124.6, 112.6, 111.6, 87.5. These data are consistent with those previously reported in the literature for this compound.^1^

**2-(2,4-difluorobenzylidene)malononitrile 3j**

 White crystals; Yield %; mp ^o^C; **^1^H NMR** (400 MHz, CDCl_3_) *δ*: 8.32−8.38 (m, 1H), 8.01 (s, 1H), 7.06−7.11 (m, 1H), 6.96−7.01 (m, 1H); **^13^C NMR** (100 MHz, CDCl_3_) *δ*: 166.8 (dd, *J* = 262.4 Hz, *J* = 13.0 Hz), 162.3 (dd, *J* = 262.2 Hz, *J* = 12.6 Hz), 149.9 (dd, *J* = 7.6 Hz, *J* = 2.0 Hz), 130.3 (dd, *J* = 10.8 Hz, *J* = 1.9 Hz), 116.0 (dd, *J* = 10.4 Hz, *J* = 3.8 Hz), 113.4 (dd, *J* = 22.2 Hz, *J* = 3.7 Hz), 113.2, 112.2, 105.2 (t, *J* = 25.5 Hz), 83.9.

**2-(2,4,6-trifluorobenzylidene)malononitrile 3k**

 White crystals; Yield; mp ^o^C; **^1^H NMR** (400 MHz, CDCl_3_) *δ*: 7.77 (s, 1H), 6.84−6.90 (m, 2H); **^13^C NMR** (100 MHz, CDCl_3_) *δ*: 165.6 (dt, *J* = 260.4 Hz, *J* = 15.6 Hz), 161.1 (ddd, *J* = 260.3 Hz, *J* = 15.2 Hz, *J* = 8.3 Hz), 146.9 (d, *J* = 1.5 Hz), 112.7, 110.8, 106.5, 101.9 (td, *J* = 25.9 Hz, *J* = 4.1 Hz), 91.0.

**2-(4-methylbenzylidene)malononitrile** **3l**^3^

White crystals; Yield; mp ^o^C; **^1^H NMR** (400 MHz, CDCl_3_) *δ*: 7.80−7.82 (m, 2H), 7.72 (s, 1H), 7.33−7.35 (m, 2H), 2.46 (s, 3H); **^13^C NMR** (100 MHz, CDCl_3_) *δ*: 159.7, 146.4, 130.9, 130.3, 128.4, 114.0, 112.8, 81.1, 22.0. These data are consistent with those previously reported in the literature for this compound.^3^

**2-(3,5-dimethylbenzylidene)malononitrile 3m**^4^

White solid; Yield; mp ^o^C; **^1^H NMR** (400 MHz, CDCl_3_) *δ*: 7.69 (s, 1H), 7.51 (m, 2H), 7.26 (s, 2H), 2.38 (m, 6H); **^13^C NMR** (100 MHz, CDCl_3_) *δ*: 160.3, 139.4, 136.6, 130.9, 128.5, 113.9, 112.7, 81.9, 21.1. These data are consistent with those previously reported in the literature for this compound.^4^

**2-(4-isopropylbenzylidene)malononitrile 3n**^1^

Yellow oil; Yield; mp ^o^C; **^1^H NMR** (400 MHz, CDCl_3_) 7.84−7.86 (m, 2H), 7.73 (s, 1H), 7.38−7.40 (m, 2H), 3.00 (h, *J* = 6.9 Hz, 1H), 1.29 (s, 3H), 1.27 (s, 3H) ; **^13^C NMR** (100 MHz, CDCl_3_) *δ*: 159.7, 157.0, 131.1, 128.7, 127.8, 114.0, 112.9, 81.2, 34.5, 23.4. These data are consistent with those previously reported in the literature for this compound.^1^

**2-(4-tertbutylbenzylidene)malononitrile 3o**^5^

 White solid; Yield; mp ^o^C; **^1^H NMR** (400 MHz, CDCl_3_) *δ*: 7.84−7.88 (m, 2H), 7.74 (s, 1H), 7.54−7.57 (m, 2H), 1.35 (s, 3H); **^13^C NMR** (100 MHz, CDCl_3_) *δ*: 159.7, 159.3, 130.9, 128.4, 126.7, 114.0, 112.9, 81.3, 35.5, 30.9. These data are consistent with those previously reported in the literature for this compound.^5^

**References:**

1. Kolagkis, P. X.; Stamatis, K. S.; Naya, A. S.; Vera, P. D.; Efthymios, T.; Eirini, M. G.; Olga, G. M.; Elpida, S.; Christoforos, G. K. Deciphering the Knoevenagel condensation: towards a catalyst-free and water-mediated process,  *Org. Biomol. Chem.* **2024**, *22*, 8293– 8299.  DOI: 10.1039/D4OB01420K
2. Zhao, X.-L.; Yang, K.; Zhang, Y.; Zhu, J.; Xu, L. Sevelamer as an Efficient and Reusable Heterogeneous Catalyst for the Knoevenagel Reaction in Water. *Chin. Chem. Lett.* **2014**, *25*, 1141.  DOI: 10.1016/j.cclet.2014.03.002
3. Emil Krogh Nielsen, Kassem El‐Chami, Clara Mondrup de Lichtenberg, Robert Madsen. Decatungstate‐Catalyzed Carbon‐Carbon Bond Formation Between Furfural and Electron‐Deficient Olefins. *European Journal of Organic Chemistry* **2024**, *27,* 16. [DOI: 10.1002/ejoc.202400109](https://doi.org/10.1002/ejoc.202400109).
4. [Chen, J.,](https://www.reaxys.com/null) [Gan, Z](https://www.reaxys.com/null)., [Zhang, Y.](https://www.reaxys.com/null), [Chen,](https://www.reaxys.com/null) Z., [Liu, S., Cui, R.,](https://www.reaxys.com/null) [Xue, Z.](https://www.reaxys.com/null), Sun, H., Lei, S.,   [Jiang, W.-F.,](https://www.reaxys.com/null) [Jin, Y. Iron-Catalyzed Photoredox Alcohol α-C–H Alkylation and Tandem Intramolecular Cyclization: Facile Access to Multisubstituted 2,3-Dihydrofurans and γ-Butyrolactones.](https://www.reaxys.com/null) *Org. Lett.* **2024**, 26, 25, 5329–5334. DOI: [10.1021/acs.orglett.4c01719](https://doi.org/10.1021/acs.orglett.4c01719).
5. Khatun, N., Kim, M. J., Kook Woo, S., Visible-Light Photoredox-Catalyzed Hydroalkoxymethylation of Activated Alkenes Using α-Silyl Ethers as Alkoxymethyl Radical Equivalents, *Org. Lett.* 2018, 20, 19, 6239–6243. DOI: 10.1021/acs.orglett.8b02721.

**^1^H and ^13^C NMR spectra of prepared compounds**

^1^H NMR (400 MHz, CDCl_3_) **3b**^1^

^13^C NMR (100 MHz, CDCl_3_) **3b**^1^

^1^H NMR (400 MHz, CDCl_3_) **3c**^2^

^13^C NMR (100 MHz, CDCl_3_) **3c**^2^

^1^H NMR (400 MHz, CDCl_3_) **3d**^1^

^13^C NMR (100 MHz, CDCl_3_) **3d**^1^

^1^H NMR (400 MHz, CDCl_3_) **3e**^1^

^13^C NMR (100 MHz, CDCl_3_) **3e**^1^

^1^H NMR (400 MHz, CDCl_3_) **3f**^1^

^13^C NMR (100 MHz, CDCl_3_) **3f**^1^

^1^H NMR (400 MHz, CDCl_3_) **3g**^1^

^13^C NMR (100 MHz, CDCl_3_) **3g**^1^

^1^H NMR (400 MHz, CDCl_3_) **3h**^1^

^13^C NMR (100 MHz, CDCl_3_) **3h**^1^

^1^H NMR (400 MHz, CDCl_3_) **3i**^1^

^13^C NMR (100 MHz, CDCl_3_) **3i**^1^

^1^H NMR (400 MHz, CDCl_3_) **3j**

^13^C NMR (100 MHz, CDCl_3_) **3j**

^1^H NMR (400 MHz, CDCl_3_) **3k**

^13^C NMR (100 MHz, CDCl_3_) **3k**

^1^H NMR (400 MHz, CDCl_3_) **3l**^3^

^13^C NMR (100 MHz, CDCl_3_) **3l**^3^

^1^H NMR (400 MHz, CDCl_3_) **3m**^4^

^13^C NMR (100 MHz, CDCl_3_) **3m**^4^

^1^H NMR (400 MHz, CDCl_3_) **3n**^1^

^13^C NMR (100 MHz, CDCl_3_) **3n**^1^

^1^H NMR (400 MHz, CDCl_3_) **3o**^5^

^13^C NMR (100 MHz, CDCl_3_) **3o**^5^
